# Relative validity of the online Meal-based Diet History Questionnaire for evaluating the overall diet quality and quality of each meal type in Japanese adults

**DOI:** 10.1017/S000711452200352X

**Published:** 2023-08-28

**Authors:** Kentaro Murakami, Nana Shinozaki, M. Barbara E. Livingstone, Nana Kimoto, Shizuko Masayasu, Satoshi Sasaki

**Affiliations:** 1 Department of Social and Preventive Epidemiology, School of Public Health, University of Tokyo, Tokyo 113-0033, Japan; 2 Nutrition Innovation Centre for Food and Health (NICHE), School of Biomedical Sciences, Ulster University, Coleraine BT52 1SA, UK; 3 Ikurien-Naka, Ibaraki 311-0105, Japan

**Keywords:** Meal type, Dietary assessment, Validity, Diet quality, Time of day of intake

## Abstract

The aim of this study was to examine the relative validity of the online Meal-based Diet History Questionnaire (MDHQ) for assessing the overall diet quality and quality of each meal type (breakfast, lunch, dinner and snacks). In total, 222 Japanese adults (111 for each sex) aged 30–76 years completed the online MDHQ and then the 4-non-consecutive-day weighed dietary record (DR). The diet quality was assessed using the Healthy Eating Index-2015 (HEI-2015) and Nutrient-Rich Food Index 9.3 (NRF9.3). For the HEI-2015, compared with the DR, the MDHQ provided high median values for breakfast (in women only) and dinner and low median values for snacks. There were no significant differences observed for overall diet and lunch. For the NRF9.3, the MDHQ provided higher median values for breakfast and dinner and a lower median value for overall diet than the DR in women, with no significant differences for lunch and snacks. In men, no significant difference was observed, except for overall diet (the MDHQ providing a lower median value). For the HEI-2015, median Spearman correlation coefficient was 0·43, with a range from 0·12 (snacks in women) to 0·68 (breakfast in men). For the NRF9.3, median Spearman correlation coefficient was 0·47, with a range from 0·26 (snacks in men) to 0·65 (breakfast in men). Bland–Altman plots showed wide limits of agreement and, in some cases, proportional bias. In conclusion, the online MDHQ showed an acceptable ability for ranking individuals according to the quality of overall diet, breakfast, lunch and dinner, but not snacks.

Sub-optimal dietary intake is a widely acknowledged major risk factor for promoting morbidity and premature death, and the improvement of diet quality is now a global priority^(1)^. An accurate assessment of habitual dietary intake is a cornerstone for identifying the diet–disease relationships and for promoting favourable changes in dietary behaviours^(2)^. Traditional nutritional epidemiological research has focused mainly on the associations between health outcomes and the intake of individual nutrients or foods, but examining the associations between health outcomes and overall dietary patterns or overall diet quality has gradually been more priority^(3)^. An increasing number of studies have evaluated the dietary intake and quality of specific eating occasions or meal patterns^(4–6)^. Examining the dietary intake and quality at each meal level to assess the overall diet may be more pertinent when considering the synergies and interactions during digestion and metabolism^(7)^.

However, research in this area has been impeded by the fact that the primary method of dietary assessment currently employed in most cross-sectional and prospective cohort studies is the FFQ, which generally precludes an informed evaluation of the timing of dietary intake and meal-specific dietary intake^(8)^. This type of information can be derived using more detailed dietary assessment methods^(9–13)^, such as dietary record (DR) and 24-h dietary recall^(4)^. However, when using these methods, the collection of dietary data for multiple days is essential for the assessment of habitual intake at the individual level, but it is not always feasible because of its expensive and burdensome nature^(14)^, despite the advancement of technology^(15)^. To our knowledge, there are no purpose-built, dedicated dietary assessment questionnaires to collect data on dietary intake at each meal type, which are also inexpensive to implement and less burdensome for participants. In this context, we recently developed the Meal-based Diet History Questionnaire (MDHQ), a self-administered questionnaire designed to estimate the dietary intake for each meal type (breakfast, lunch, dinner and snacks) separately^(16,17)^.

The MDHQ has several advantages. First, the MDHQ is data-driven, so the development of the questionnaire structure, food items and dietary intake calculation algorithms was based on detailed dietary information derived from the 16-d weighed DR obtained from 242 Japanese adults^16^. Second, because the MDHQ assesses dietary intake for each meal type separately and given that the cognitive tasks required during dietary recall are complex^(18)^, the MDHQ may be easier to complete, facilitating better estimation of food intake. This may be particularly relevant to the Japanese because previous studies of Japanese adults have shown that the selection, amount and combination of foods consumed are markedly different between meal types^(6,19–22)^. Third, the MDHQ provides information on various aspects of dietary behaviours, such as breakfast quality and percentage of energy from snacking occasions. However, a rigorous evaluation of the validity of the MDHQ has not been conducted yet, except for food group intake^(23)^.

The primary aim of this study was to examine the relative validity of the web version of the MDHQ for assessing the overall diet quality and quality of each meal type (breakfast, lunch, dinner and snacks). In the real world, not all study participants would complete the questionnaire online. Thus, the secondary aim was to similarly examine the relative validity of the paper version of the MDHQ. The diet quality was assessed using the Healthy Eating Index-2015 (HEI-2015)^(24–27)^ and Nutrient-Rich Food Index 9.3 (NRF9.3)^(27–31)^.

## Methods

### Study procedure and participants

This cross-sectional study was based on the data collected from fourteen (of the forty-seven) prefectures between August and October 2021. Recruitment of participants and data collection were conducted by our research dietitians (*n* 60) with expertise in collecting DR data^(32,33)^. First, healthy women aged 30–69 years who were willing to participate and were living with their husbands were recruited for this study. For each prefecture, two women from each 10-year age category (30–39, 40–49, 50–59 and 60–69 years) were selected. Their husbands were then recruited (irrespective of age), resulting in 112 individuals by sex. The sample size was determined primarily based on the recommendation made by Cade et al. that for validation studies, a sample size of at least 50 and preferably much larger (e.g. 100 or more subjects) is desirable^(14)^. To minimise the dropout rate, the potential participants were restricted to individuals who had full understanding of the procedure and showed willingness to complete the entire survey. Meanwhile, dietitians, individuals living with a dietitian, those who had received dietary counselling from a doctor or dietitian, those taking insulin treatment for diabetes, those undergoing dialysis treatment, those without sufficient Internet access, those who had difficulty answering the web-based questionnaires and pregnant or lactating women were excluded. Only participation in the study as a couple (one woman and one man) was permitted.

**Fig. 1.**
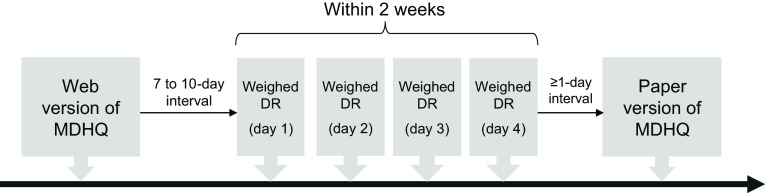
Study schedule. MDHQ, Meal-based Diet History Questionnaire; DR, dietary record.

The study schedule is shown in Fig. 1. Each participant was asked to answer the web version of the MDHQ (web MDHQ). After an interval of 7–10 d (to ensure the completion of the web MDHQ), a 4-non-consecutive-day weighed DR was conducted for 2 weeks. Finally, after an interval of at least 1 d, the paper version of the MDHQ (paper MDHQ) was completed. We designed this schedule because the main purpose of this study was to evaluate the validity of the web MDHQ; thus, a web MDHQ survey was performed prior to the conduct of DR. A total of 111 women aged 30–69 years and 111 men aged 30–76 years completed the study. As a financial incentive, each couple received a voucher worth 5000 Japanese Yen (31 British Pound as of 1 October 2022) after the study.

The study was conducted in accordance with the guidelines of the Declaration of Helsinki, and all procedures involving humans were approved by the Ethics Committee of the University of Tokyo Faculty of Medicine (protocol code: 2020326NI; date of approval: 29 January 2021). Written informed consent was obtained from all participants.

### Meal-based Diet History Questionnaire

Details of the MDHQ have been published elsewhere^(16,17)^. Briefly, the MDHQ is a self-administered questionnaire designed to estimate dietary intake in the previous month for each meal type (breakfast, morning snack, lunch, afternoon snack, dinner and night snack). The MDHQ comprises three parts. Part 1 of the MDHQ includes quantitative questions on the consumption frequency of generic food groups (Tier 1 food groups) for each meal type, with potential answers of 0–7 d/week. Part 2 of the MDHQ includes questions on the relative consumption frequency of sub-food groups (Tier 2 food group) within one of the generic food groups (Tier 1 food group), with possible answers of ‘always, often, sometimes, rarely, and never’. By combining the information derived from Parts 1 and 2, the number of foods that can be estimated efficiently can be increased but within a limited number of questions. Part 3 of the MDHQ enquires about the general eating behaviours, including the amount of brown rice consumed, the relative consumption frequency of wholegrain bread and whether bread was consumed with jam, honey, etc. or with fat spread. Finally, the MDHQ includes the assessment of basic characteristics (sex, age, body height, body weight, education level and current smoking status).

In the MDHQ, information on portion sizes was not collected (except for alcoholic beverages, for which the overall consumption frequency and portion size were assessed in Part 2). This decision was based on our previous observation that the Brief-type Diet History Questionnaire (BDHQ), which assesses the consumption frequency of fifty-eight food items but does not collect information on portion sizes and applies fixed portion sizes for dietary intake calculation, had a similar efficacy in estimating the food and nutrient intake as the Diet History Questionnaire (DHQ), which assesses not only the consumption frequency but also the portion size of 150 food items^(27,34,35)^. The limited usefulness of portion size information has also been supported by several previous studies^(36,37)^. All the food groups included in the MDHQ (see online Supplementary Table S1), as well as the sex-specific and meal-type-specific portion sizes, were determined based on the 16-d weighed DR data collected from 121 Japanese women and 121 Japanese men, comprising 206 837 food item entries^(16)^.

In the present study, two delivery modes of MDHQ, which are identical in terms of content, were used: web MDHQ and paper MDHQ. The web MDHQ was prepared using Google Forms. Each question was answered by each participant, with non-response not permitted. All responses to the web MDHQ automatically allocated into a spreadsheet format were downloaded from Google Drive. The paper MDHQ used in this study was an A4 21-page questionnaire. Responses to all questions were checked by the research dietitians and staff at the study centre. If any responses were missing, the participants were asked to answer the questions again in person or by phone. All answers in the paper MDHQ were manually entered into a spreadsheet in duplicate, and any discrepancies were checked and corrected. Data obtained using the web MDHQ and paper MDHQ were converted to a dataset suitable for dietary intake calculation.

On the basis of a series of *ad hoc* computer algorithms in the MDHQ^(16)^, estimated intakes of Tier 1 and 2 food groups were calculated. Estimated intakes of energy and nutrients were calculated using food intake information and the 2015 version of the Standard Tables of Food Composition in Japan^(38)^. Component scores needed for the calculation of HEI-2015 were calculated using the Japanese version^(27)^ of the US Food Patterns Equivalents Database^(39)^. These calculations were done for each meal type, and the overall intake was calculated as the sum of the intake of each meal type.

### Four-day weighed dietary record

The 4-non-consecutive-day weighed DR was selected as the reference method in this validation study. Each recording period consisted of three weekdays (Monday–Friday, except for national holidays) and one weekend day (Saturday, Sunday or national holidays). For each couple, a recording day was allocated within 2 weeks by research dietitians. Each couple was provided with recording sheets and a digital scale (KS-274, Dretec, Japan; ±2 g precision for 0–500 g and ±3 g precision for 500–2000 g). After receiving written and verbal instructions from the assigned research dietitian, as well as an example of a completed diary sheet, each participant was requested to document and weigh all consumed foods and drinks, both inside and outside of their homes, on each recording day. On certain occasions when weighing was inconvenient to carry out (e.g. dining out), they were instructed to document as much information as possible, including the brand name of the food and the consumed portion size (based on typical household measures), as well as the details of the leftovers.

The recording sheets used in each survey day were submitted directly to the research dietitian after the survey was completed, who then reviewed the forms and, whenever necessary, sought additional information or modified the record via phone or in-person interview. All collected records were then reviewed by the research dietitians and trained staff at the study centre. In accordance with a standardised procedure, the portion sizes estimated using household measures were converted into weights, and the individual food items were coded based on the 2015 version of the Standard Tables of Food Composition in Japan^(38)^. A total of 1297 food codes were used in the DR.

The structure of the food diary sheet used was based on a typical Japanese eating pattern, which comprised breakfast, lunch, dinner and snacks; these meal types were prescribed in the diary. For DR data, the name of the meal type used in the present analysis was based on this classification. As was the case in the MDHQ, estimated intakes of energy and nutrients and component scores needed for the calculation of HEI-2015 were calculated using the 2015 version of the Standard Tables of Food Composition in Japan^(38)^ and the Japanese version^(27)^ of the US Food Patterns Equivalents Database^(39)^, respectively. These calculations were done for each meal type, and the overall intake was calculated as the sum of the intake of each meal type. For all dietary variables, the mean daily values within the 4-d period were used for each individual.

### Healthy Eating Index-2015

As described elsewhere^(24–27)^, HEI-2015 is a composite measure of compliance with the 2015–2020 Dietary Guidelines for Americans^(40)^. The HEI-2015 is a 100-point scale, with a higher score indicating a better quality of diet. The HEI-2015 consists of nine adequacy components, namely, total fruits (maximum score: 5), whole fruits (5), total vegetables (5), greens and beans (5), whole grains (10), dairy products (10), total protein foods (5), seafood and plant proteins (5) and fatty acids as the ratio of the sum of PUFA and MUFA to SFA (10), and four moderation components, namely, refined grains (10), Na (10), added sugars (10) and saturated fats (10). For each meal type and overall diet for each participant for each dietary assessment method, we calculated the HEI-2015 component and total scores based on energy-adjusted values of dietary intake, namely, amount per 4184 kJ (1000 kcal) of energy or percentage of energy, except for the fatty acids component^(27)^.

### Nutrient-rich Food Index 9.3

The overall diet quality was also assessed using the NRF9.3, as described in detail elsewhere^(27–31)^. The NRF9.3 is a composite measure of the nutrient density of the diet, calculated as the sum of the percentage of reference daily values for nine qualifying nutrients, namely, protein, dietary fibre, vitamin A, vitamin C, vitamin D, Ca, Fe, K and Mg, minus the sum of the percentage of reference daily values for three disqualifying nutrients, namely, added sugars, saturated fats and Na. Reference daily values were determined for sex and age categories, based on the Dietary Reference Intakes for Japanese, 2020^(41)^, namely, the RDA for protein, vitamin A, vitamin C, Ca, Fe and Mg and tentative dietary goal for preventing lifestyle-related diseases for dietary fibre, K, saturated fats and Na. For added sugars, the conditional recommendation advocated by the WHO (i.e. upper limit of 5 % of energy)^(42)^ was used because of the lack of a recommended value for added sugars in Japan, as well as their low intake levels^(43)^. We calculated the NRF9.3 component and total scores based on the daily intake of each nutrient for each participant, which was adjusted for energy intake by the density method and then normalised for the sex- and age-specific Estimated Energy Requirement for a moderate level of physical activity (from the Dietary Reference Intakes for Japanese, 2020^(41)^) and expressed as a percentage of the reference daily value^(27)^. These calculations were done for each meal type and for overall diet. Higher NRF9.3 scores indicated a better quality of the diet. A maximum possible score of 900 indicated a diet in which intakes per given amount of energy were above the reference daily values for the nine qualifying nutrients but below the reference daily values for the three disqualifying nutrients. In this study, dietary supplements were not considered during the nutrient intake calculation in any of the dietary assessment methods because it was our intention to assess nutrient intake from foods and beverages only.

### Statistical analysis

Statistical analyses were performed using the SAS statistical software (version 9.4; SAS Institute Inc.). A two-tailed *P* value of < 0·05 was considered significant. Analyses were stratified by sex and conducted to determine the overall intake and intake for each meal type (breakfast, lunch, dinner and snacks). The dietary variables examined in this study included the total and component scores of HEI-2015 and NRF9.3, in addition to energy intake (MJ/d) and percentage of energy intake from each meal type. The amounts of snacks consumed were combined for analysis due to their relatively low intake in both methods. All dietary data were expressed as median and 25th and 75th percentiles. To assess the estimation ability at the group level, the median values of estimates derived from the MDHQ were compared with those derived from the DR using the Wilcoxon signed-rank test. The Spearman correlation coefficients between the MDHQ and DR estimates were used to assess the ability of the MDHQ to rank individuals in a population. In addition, agreement of the total scores of HEI-2015 and NRF9.3 between the MDHQ and DR was assessed using the Bland–Altman analysis^(44)^. To examine the proportional bias between the MDHQ and DR, the Bland–Altman analysis was accompanied by the linear regression analysis^(45)^. Identical analyses were conducted to assess the web MDHQ and paper MDHQ. The findings (tables and figures) on the web MDHQ are provided in the “Results” section, whereas those on the paper MDHQ are provided as online Supplementary Materials.

## Results

This study included 111 women and 111 men aged 30–69 years and 30–76 years, respectively (Table 1). The median BMI (in kg/m^2^) was 22·0 for women and 23·5 for men.

**Table 1.** Basic characteristics of the study population

	Women (*n* 111)				Men (*n* 111)			
	Median	P25, P75	*n*	%	Median	P25, P75	*n*	%
Age (years)	50·0	39·0, 60·0			50·0	41·0, 62·0		
Body height (cm)*	157·5	155·0, 163·0			170·0	165·8, 174·5		
Body weight (kg)*	56·0	50·2, 62·5			68·0	60·0, 76·0		
BMI (kg/m^2^)†	22·0	20·3, 24·9			23·5	21·1, 26·1		
Education level								
Junior high school or high school			28	25·2			41	36·9
College or technical school			55	49·5			22	19·8
University or higher			28	25·2			48	43·2
Current smoking status								
Smoker			12	10·8			35	31·5
Non-smoker			99	89·2			76	68·4

P25, 25th percentile; P75, 75th percentile.

* Based on self-report.

† Calculated using the self-reported body height and weight.

### Results on the web version of Meal-based Diet History Questionnaire

#### Median estimations

The median estimates of the total and component scores of HEI-2015 and NRF9.3, energy intake and percentage of energy intake derived from the DR and web MDHQ are shown in Table 2 for women and Table 3 for men, according to the meal type. The number of HEI-2015 components (*n* 13 in total) showing no significant differences in women was 8 for overall diet, 8 for breakfast, 10 for lunch, 4 for dinner and 6 for snacks. The corresponding number in men was 5, 8, 9, 4 and 5, respectively. The number of NRF9.3 components (*n* 12 in total) showing no significant differences in women was 6 for overall diet, 3 for breakfast, 8 for lunch, 7 for dinner and 11 for snacks. The corresponding number in men was 7, 9, 6, 4 and 8, respectively.

**Table 2.** Median estimates of the total and component scores of Healthy Eating Index-2015 (HEI-2015) and Nutrient-Rich Food Index 9.3 (NRF-9.3), energy intake and percentage of energy intake derived from the 4-d weighed dietary record (DR) and those derived from the web version of the Meal-based Diet History Questionnaire (MDHQ) in 111 Japanese women, according to meal type*

	Overall diet				Breakfast				Lunch				Dinner				Snacks			
	DR		Web MDHQ		DR		Web MDHQ		DR		Web MDHQ		DR		Web MDHQ		DR		Web MDHQ	
	Median	P25, P75	Median	P25, P75	Median	P25, P75	Median	P25, P75	Median	P25, P75	Median	P25, P75	Median	P25, P75	Median	P25, P75	Median	P25, P75	Median	P25, P75
HEI-2015†	51·3	45·5, 57·0	50·3	45·7, 56·3	44·2	36·6, 51·3	**49·9 b**	42·1, 55·8	46·8	39·4, 52·3	48·0	41·6, 52·7	53·0	47·0, 57·4	**56·1 c**	51·2, 61·9	40·0	31·0, 46·7	**31·1 c**	23·3, 38·5
Total fruits	1·4	0·5, 2·8	1·7	0·6, 3·1	1·0	0, 5·0	1·1	0·1, 4·2	0·4	0, 1·7	0·4	0, 2·2	0	0, 1·1	**0·9 c**	0, 2·4	0·3	0, 4·8	1·2	0·1, 4·6
Whole fruits	2·4	0·9, 5·0	3·1	1·1, 5·0	1·9	0, 5·0	1·8	0·1, 5·0	0·8	0, 3·3	0·4	0, 4·2	0	0, 2·3	**1·6 c**	0, 4·6	0	0, 5·0	**1·2 a**	0·1, 5·0
Total vegetables	5·0	4·1, 5·0	5·0	4·3, 5·0	2·3	0, 4·9	**3·5 b**	0·2, 5·0	4·4	3·1, 5·0	4·8	2·2, 5·0	5	5·0, 5·0	5·0	5, .0 5·0	0	0, 1·2	0·4	0·2, 0·9
Greens and beans	1·6	0·5, 3·5	**2·4 a**	1·4, 3·7	0	0, 1·6	**0·9 c**	0·1, 3·1	0·3	0, 3·3	1·7	0·3, 2·9	1·2	0, 4·4	**4·1 c**	1·8, 5·0	0	0, 0	**0·2 c**	0, 0·5
Whole grains	0	0, 0·4	**0·8 c**	0·1, 1·6	0	0, 0	**1·1 c**	0, 3·8	0	0, 0	**0·3 c**	0, 1·6	0	0, 0	**0·1 c**	0, 0·8	0	0, 0	0	0, 0
Dairy	2·3	1·1, 4·3	2·6	1·4, 4·0	4·0	0·3, 9·5	**5·9 b**	1·2, 10	0·5	0, 2·0	0·6	0, 2·2	0·2	0, 1·3	0·5	0, 1·2	2·7	0·1, 9·2	**1·1 b**	0·5, 4·4
Total protein foods	5·0	5·0, 5·0	**4·9 c**	3·9, 5·0	4·5	1·2, 5·0	4·1	2·3, 5·0	4·9	3·8, 5·0	**4·4 c**	2·3, 5·0	5·0	5·0, 5·0	5·0	5·0, 5·0	0·3	0, 2·1	**0·3 b**	0·2, 0·5
Seafood and plant proteins	5·0	5·0, 5·0	5·0	4·7, 5·0	4·4	0, 5·0	4·6	0, 5·0	4·6	2·2, 5·0	3·6	1·4, 5·0	5·0	5·0, 5·0	**5·0 a**	5, .0 5·0	0	0, 3·4	0·4	0·2, 0·6
Fatty acids‡	6·0	3·5, 8·4	5·7	3·7, 7·6	2·6	0, 7·4	4·1	0·5, 7·5	8·2	4·5, 10	7·5	4·4, 10	9·6	6, 10	**10 c**	9·5, 10	0	0, 1·7	**0 b**	0, 0
Refined grains	1·4	0, 3·8	0·7	0, 3·2	0	0, 3·0	0	0, 2·3	0	0, 0·9	0	0, 0	4·1	1·4, 9·6	**3·1 a**	0·8, 7·5	10	5·3, 10	**4·8 c**	1·3, 8·4
Na	0·5	0, 4·4	**0 c**	0, 1·4	4·7	0, 10	**2·1 c**	0, 8·0	0	0, 3·1	0	0, 6·0	0	0, 3·1	**0 c**	0, 0	10	10, 10	10	10, 10
Added sugars	9·8	8·7, 10	**9·5 b**	7·8, 10	10	8·1, 10	10	8·0, 10	10	9·3, 10	10	9·7, 10	10	10, 10	10	10, 10	0·8	0, 6·4	**0 b**	0, 3·2
Saturated fats	8·4	6·6, 10	8·7	7·2, 10	7·2	4·0, 10	7·6	5·3, 10	10	8·1, 10	**10 b**	8·8, 10	8·9	7·1, 10	**10 b**	8·8, 10	6·0	0, 10	4·1	0, 9·2
NRF9.3§	606	511, 677	**585 a**	489, 658	507	399, 610	**590 b**	442, 680	493	411, 628	534	401, 636	634	515, 711	**661 a**	589, 706	1	–284, 321	–1	–218, 192
Protein	100	100, 100	100	100, 100	100	100, 100	**100 a**	100, 100	100	100, 100	**100 c**	100, 100	100	100, 100	100	100, 100	80	60, 100	**69 b**	60, 80
Dietary fibre	77	65, 88	**74 a**	62, 84	76	58, 100	76	62, 94	76	57, 91	**70 a**	57, 84	85	66, 100	86	68, 100	45	18, 61	48	32, 55
Vitamin A	55	45, 75	**67 c**	50, 83	56	30, 76	**64 c**	44, 82	53	33, 73	55	30, 84	57	42, 87	**85 c**	64, 100	38	9, 65	34	26, 46
Vitamin C	86	67, 100	90	70, 100	49	23, 100	**84 c**	42, 100	69	44, 100	79	43, 100	100	76, 100	100	82, 100	33	5, 100	53	13, 100
Vitamin D	68	41, 100	59	47, 82	36	17, 62	**49 c**	30, 79	31	16, 68	37	23, 66	86	33, 100	**99 b**	57, 100	10	2, 26	18	12, 23
Ca	81	62, 100	84	71, 100	94	61, 100	**100 b**	78, 100	66	44, 88	57	43, 84	65	49, 84	69	56, 80	100	73, 100	90	73, 100
Fe	95	69, 100	89	69, 100	81	50, 100	**89 a**	58, 100	84	64, 100	**75 c**	58, 100	100	75, 100	99	76, 100	66	42, 100	69	52, 94
K	99	84, 100	**100 b**	92, 100	99	69, 100	**100 c**	95, 100	76	63, 96	81	62, 97	100	88, 100	100	93, 100	100	80, 100	99	78, 100
Mg	93	82, 100	**100 c**	91, 100	100	73, 100	**100 c**	92, 100	74	62, 96	83	67, 97	100	86, 100	**100 b**	94, 100	93	61, 100	85	75, 100
Added sugars	40	0, 83	**51 b**	0, 120	0	0, 109	12	0, 112	0	0, 56	0	0, 42	0	0, 0	0	0, 0	401	163, 713	456	299, 642
Saturated fats	34	17, 56	30	7, 50	48	1, 86	45	11, 72	14	0, 40	**0 b**	0, 31	29	1, 50	**13 c**	0, 31	63	0, 144	86	26, 134
Na	51	23, 71	69 c	42, 98	21	0, 58	**36 c**	1, 106	68	33, 109	60	12, 116	71	37, 105	**100 c**	69, 138	0	0, 0	0	0, 0
Energy intake (MJ/d)	7·4	6·3, 8·1	**6·0 c**	5·3, 7·2	1·6	1·3, 2·0	**1·5 c**	1·1, 1·8	2·2	1·9, 2·6	**1·5 c**	1·3, 1·9	2·6	2·1, 3·2	**2·1 c**	1·8, 2·4	0·6	0·3, 1	**0·9 c**	0·5, 1·4
Percentage of energy intake	–	–	–	–	23·1	17·8, 26·6	24·0	18·9, 29·0	31·2	26·8, 36·2	**25·5 c**	20·9, 30·4	36·4	31·8, 41·1	**34·8 a**	30·3, 39·7	8·7	4·4, 14·6	**13·8 c**	8·7, 21·6

P25, 25th percentile; P75, 75th percentile.

* The values derived from the MDHQ were compared with those derived from the DR using the Wilcoxon signed-rank test (a: *P* < 0·05, b: *P* < 0·01 and c: *P* < 0·001; shown in bold).

† Calculated as the sum of all components scores. A maximum score is 100. A maximum score for each component is as follows: 5 for total fruits, whole fruits, total vegetables, greens and beans, total protein foods, and seafood and plant proteins and 10 for whole grains, dairy products, fatty acids, refined grains, Na, added sugars and saturated fats. A higher score indicates a higher diet quality (i.e. a lower intake for refined grains, Na, added sugars, and saturated fats components and a higher intake for other components).

‡ Defined as the ratio of the sum of PUFA and MUFA to SFA.

§ Calculated as the sum of scores for nine nutrients to encourage (i.e. protein, dietary fibre, vitamins A, C and D, Ca, Fe, K and Mg) minus the sum of scores for three nutrients to limit (i.e. added sugars, saturated fats and Na). A maximum score is 900. For each component, a maximum score is 100, except for added sugars, saturated fats, and Na components, for which a maximum score is infinite depending on the intake level. A higher score indicates a higher diet quality, except for added sugars, saturated fats and Na components, for which a higher score indicates an unfavourable dietary intake (i.e. higher intakes of added sugars, saturated fats and Na).

**Table 3.** Median estimates of the total and component scores of Healthy Eating Index-2015 (HEI-2015) and Nutrient-Rich Food Index 9.3 (NRF9.3), energy intake and percentage of energy intake derived from the 4-d weighed dietary record (DR) and those derived from the web version of the Meal-based Diet History Questionnaire (MDHQ) in 111 Japanese men, according to meal type*

	Overall diet				Breakfast				Lunch				Dinner				Snacks			
	DR		Web MDHQ		DR		Web MDHQ		DR		Web MDHQ		DR		Web MDHQ		DR		Web MDHQ	
	Median	P25, P75	Median	P25, P75	Median	P25, P75	Median	P25, P75	Median	P25, P75	Median	P25, P75	Median	P25, P75	Median	P25, P75	Median	P25, P75	Median	P25, P75
HEI-2015†	49·5	44·7, 54·5	48·6	43·4, 55·4	43·2	36·8, 50·9	44·6	39·1, 51·3	43·3	36·0, 49·2	44·5	38·0, 51·5	51·9	46·7, 57·6	**54·3 b**	50·4, 59·4	39·2	30·0, 43·0	**33·1 b**	28·4, 40·0
Total fruits	0·7	0, 1·6	**0·8 a**	0·2, 2·2	0	0, 3·2	0·5	0, 5·0	0	0, 0·5·0	**0·1 a**	0, 1·0	0	0, 0·7	**0·4 b**	0, 1·5	0	0, 0·7	**0·3 c**	0, 3·4
Whole fruits	1·0	0, 2·9	1·0	0·3, 3·5	0	0, 5·0	0·3	0, 5·0	0	0, 0·5·0	0	0, 1·2	0	0, 1·2	**0·7 a**	0, 2·2	0	0, 0	**0·1 c**	0, 5·0
Total vegetables	5·0	3·4, 5·0	**4·7 b**	2·9, 5·0	1·4	0, 5·0	2·2	0, 4·9	3·7	2·3, 5·0	**3·3 b**	0·8, 5·0	5·0	5·0, 5·0	**5·0 b**	4·4, 5·0	0	0, 0·3	**0·3 b**	0, 1·0
Greens and beans	1·4	0·2, 2·9	1·4	0·7, 2·6	0	0, 0·6	**0·4 a**	0, 1·6	0·1	0, 1·5	0·7	0·1, 1·5	1·1	0, 3·8	**2·3 b**	0·7, 4·5	0	0, 0	**0·1 c**	0, 0·4
Whole grains	0	0, 0	**0·3 c**	0, 1·7	0	0, 0	**0 c**	0, 0	0	0, 0	**0 b**	0, 1·3	0	0, 0	**0 c**	0, 0·6	0	0, 0	0	0, 0
Dairy	1·3	0·5, 2·9	**1·3 a**	0·3, 2·4	1·7	0, 7·9	0·8	0, 9·5·0	0·2	0, 0·9	0·1	0, 0·8	0·1	0, 1·3	0·1	0, 0·7	0·6	0, 4·4	**0·8 a**	0·2, 1·7
Total protein foods	5·0	4·9, 5·0	**4·5 c**	3·7, 5·0	4·8	2·2, 5·0	**3·9 b**	1·2, 5·0	4·9	3·3, 5·0	4·5	3·0, 5·0	5·0	5·0, 5·0	**5·0 a**	5·0, 5·0	0	0, 1·1	**0·2 a**	0, 0·5
Seafood and plant proteins	5·0	5·0, 5·0	5·0	4·5, 5·0	4·3	0, 5·0	**2·1 a**	0, 5·0	3·3	1·4, 5·0	4·0	1·1, 5·0	5·0	5·0, 5·0	5·0	5·0, 5·0	0	0, 0·5	0·2	0, 0·6
Fatty acids‡	6·3	4·7, 8·6	6·9	4·9, 9·2	3·8	0, 8·3	4·2	1, 7·4	7·6	4·4, 10	7·5	4·5, 10	8·9	6·1, 10	**10 c**	9·6, 10	0	0, 1·9	0	0, 0·9
Refined grains	0·8	0, 2·7	**0 b**	0, 2·7	0	0, 2·7	0	0, 3·0	0	0, 0	0	0, 0	3·7	0, 9·3	**3·0 a**	0, 6·9	10	8·0, 10	**7·3 c**	2·2, 10
Na	1·5	0, 4·1	**0 b**	0, 2·3	4·4	0, 8·4	2·6	0, 10	0	0, 3·3	0·5	0, 5·0	1·3	0, 4·7	0	0, 2·9	10	10, 10	**10 a**	10, 10
Added sugars	10	9·1, 10	10	8·2, 10	10	7·4, 10	10	8·8, 10	10	10, 10	10	10, 10	10	10, 10	10	10, 10	2·2	0, 10	0·2	0, 7·1
Saturated fats	9·4	8, 10	**10 c**	9·4, 10	8·1	5·4, 10	**9·8 b**	6·7, 10	10	8·4, 10	**10 b**	10, 10	10	7·9, 10	**10 c**	10, 10	10	2·3, 10	10	3·3, 10
NRF9.3§	605	495, 703	**584 a**	494, 659	504	366, 649	549	382, 659	483	398, 585	540	386, 618	611	552, 720	636	559, 691	75	–258, 322	40	–184, 200
Protein	100	100, 100	**100 a**	100, 100	100	100, 100	100	100, 100	100	100, 100	100	100, 100	100	100, 100	**100 b**	100, 100	73	46, 100	**66 a**	50, 75
Dietary fibre	71	59, 88	71	59, 84	78	59, 100	81	58, 100	70	55, 92	**67 a**	54, 83	79	59, 98	78	57, 96	25	0, 50	**44 a**	19, 59
Vitamin A	48	34, 64	54	35, 70	44	26, 66	51	31, 74	45	27, 64	42	18, 67	54	33, 77	**60 a**	37, 97	13	0, 36	27	9, 41
Vitamin C	93	72, 100	94	66, 100	61	22, 100	85	28, 100	71	46, 100	75	33, 100	100	81, 100	100	69, 100	28	1, 100	43	12, 100
Vitamin D	79	50, 100	79	57, 100	44	23, 81	60	30, 92	38	22, 85	**59 a**	30, 99	97	46, 100	**100 b**	67, 100	7	0, 23	18	4, 27
Ca	72	57, 87	70	57, 85	82	54, 100	83	51, 100	54	41, 73	50	39, 69	65	48, 80	**56 a**	44, 71	100	66, 100	**81 b**	51, 100
Fe	100	100, 100	**100 c**	93, 100	100	88, 100	**100 b**	74, 100	100	90, 100	**96 b**	81, 100	100	100, 100	**100 c**	87, 100	73	32, 100	85	48, 100
K	96	81, 100	98	85, 100	100	70, 100	100	73, 100	74	59, 91	75	60, 90	100	88, 100	**100 a**	77, 100	100	72, 100	100	72, 100
Mg	88	76, 100	**92 a**	80, 100	93	71, 100	99	76, 100	66	57, 79	**76 b**	61, 86	94	79, 100	92	79, 100	96	57, 100	87	60, 100
Added sugars	0	0, 68	20	0, 110	0	0, 134	0	0, 79	0	0, 0	0	0, 13	0	0, 0	0	0, 0	330	0, 689	439	146, 667
Saturated fats	24	0, 40	**4 c**	0, 24	39	0, 72	**18 b**	0, 54	6	0, 34	**0 c**	0, 13	18	0, 42	**0 c**	0, 7	4	0, 106	19	0, 97
Na	60	43, 90	**75 c**	57, 111	42	9, 80	**54 a**	5, 118	89	45, 145	**75 a**	37, 122	67	38, 106	**82 b**	51, 140	0	0, 0	**0 a**	0, 0
Energy intake (MJ/d)	9·3	8·1, 10·9	**8·0 c**	6·4, 9·2	1·9	1·2, 2·4	**1·7 c**	1·1, 2·2	2·9	2·3, 3·4	**2·2 c**	1·8, 2·7	3·6	3·1, 4·6	**3·0 c**	2·4, 3·7	0·7	0·3, 1·3	**0·9 b**	0·4, 1·7
Percentage of energy intake	–	–	–	–	20·0	13·8, 24·2	21·5	14·0, 25·8	31·2	26·9, 33·5	**28·0 c**	22·4, 31·8	36·4	31·8, 41·1	**34·8 a**	30·3, 39·7	7·3	3·2, 13·3	**12·3 c**	5·6, 20·8

P25, 25th percentile; P75, 75th percentile.

* The values derived from the MDHQ were compared with those derived from the DR using the Wilcoxon signed-rank test (a: *P* < 0·05, b: *P* < 0·01 and c: *P* < 0·001; shown in bold).

† Calculated as the sum of all components scores. A maximum score is 100. A maximum score for each component is as follows: 5 for total fruits, whole fruits, total vegetables, greens and beans, total protein foods, and seafood and plant proteins and 10 for whole grains, dairy products, fatty acids, refined grains, Na, added sugars and saturated fats. A higher score indicates a higher diet quality (i.e. a lower intake for refined grains, Na, added sugars and saturated fats components and a higher intake for other components).

‡ Defined as the ratio of the sum of PUFA and MUFA to SFA.

§ Calculated as the sum of scores for nine nutrients to encourage (i.e. protein, dietary fibre, vitamins A, C and D, Ca, Fe, K and Mg) minus the sum of scores for three nutrients to limit (i.e. added sugars, saturated fats and Na). A maximum score is 900. For each component, a maximum score is 100, except for added sugars, saturated fats and Na components, for which a maximum score is infinite depending on the intake level. A higher score indicates a higher diet quality, except for added sugars, saturated fats and Na components, for which a higher score indicates an unfavourable dietary intake (i.e. higher intakes of added sugars, saturated fats and Na).

For the HEI-2015 total score, the web MDHQ provided higher median values for breakfast (in women only: +5·7; with no significant difference in men: +1·4) and dinner (+3·1 for women and +2·4 for men) and lower median values for snacks (–8·9 for women and –6·1 for men) than the DR, although these differences were relatively small, particularly for dinner. There were no significant differences observed for overall diet (–1·0 for women and –0·9 for men) and lunch (+1·2 for both sexes). For the NRF9.3 total score, the web MDHQ provided higher median values for breakfast (+83) and dinner (+27) and a lower median value for overall diet (–21) than the DR in women, although again the differences were relatively small, except for breakfast. No significant differences were observed for lunch (+41) and snacks (–2). In men, no significant difference was observed, except for overall diet, for which the web MDHQ provided a lower median value (–21) than the DR.

For energy intake, the web MDHQ provided lower median values for overall diet, breakfast, lunch and dinner and higher median values for snacks than the DR in both sexes. When expressed as percentage of energy intake, the web MDHQ provided higher median values for snacks and lower median values for lunch and dinner than the DR, with no significant differences for breakfast in either sex.

#### Spearman correlations

Table 4 shows Spearman correlation coefficients between estimates of the total and component scores of HEI-2015 and NRF9.3, energy intake and percentage of energy intake derived from the DR and web MDHQ. For the HEI-2015 components, median correlation coefficients were 0·39 for overall diet, 0·54 for breakfast, 0·26 for lunch, 0·28 for dinner and 0·21 for snacks in women. The corresponding values in men were 0·28, 0·55, 0·24, 0·20 and 0·22, respectively. For the NRF9.3 components, median correlation coefficients were 0·47 for overall diet, 0·41 for breakfast, 0·33 for lunch, 0·42 for dinner and 0·27 for snacks in women. The corresponding values in men were 0·45, 0·50, 0·29, 0·32 and 0·27, respectively. For the HEI-2015 total score, median correlation coefficient was 0·43, with a range from 0·12 (snacks in women) to 0·68 (breakfast in men). For the NRF9.3 total score, median correlation coefficient was 0·47, with a range from 0·26 (snacks in men) to 0·65 (breakfast in men). For energy intake variables, median correlation coefficient was 0·45, with a range from 0·27 (percentage of energy from lunch in women) to 0·65 (energy from breakfast in men).

**Table 4.** Spearman correlation coefficients between estimates of the total and component scores of Healthy Eating Index-2015 (HEI-2015) and Nutrient-Rich Food Index 9.3 (NRF9.3), energy intake and percentage of energy intake derived from the 4-d weighed dietary record (DR) and those derived from the web version of the Meal-based Diet History Questionnaire (MDHQ) in 111 Japanese women and 111 Japanese men, according to meal type*

	Women					Men				
	Overall diet	Breakfast	Lunch	Dinner	Snacks	Overall diet	Breakfast	Lunch	Dinner	Snacks
HEI-2015†	**0·49 c**	**0·53 c**	**0·43 c**	**0·40 c**	0·12	**0·57 c**	**0·68 c**	**0·43 c**	**0·37 c**	0·14
Total fruits	**0·62 c**	**0·48 c**	**0·40 c**	**0·38 c**	**0·26 b**	**0·58 c**	**0·64 c**	**0·45 c**	**0·19 a**	**0·24 b**
Whole fruits	**0·65 c**	**0·50 c**	**0·38 c**	**0·39 c**	**0·21 a**	**0·61 c**	**0·67 c**	**0·42 c**	**0·20 a**	**0·34 c**
Total vegetables	**0·37 c**	**0·66 c**	**0·35 c**	**0·39 c**	0·18	**0·48 c**	**0·54 c**	**0·24 b**	**0·40 c**	**0·31 c**
Greens and beans	**0·27 b**	**0·43 c**	**0·26 b**	**0·36 c**	0·04	0·17	**0·36 c**	0·14	0·16	**0·20 a**
Whole grains	**0·33 c**	**0·31 b**	0·10	**0·26 b**	Not available	**0·34 c**	**0·30 b**	**0·23 a**	**0·43 c**	Not available
Dairy	**0·50 c**	**0·55 c**	0·09	0·18	**0·43 c**	**0·59 c**	**0·57 c**	**0·43 c**	0·09	**0·21 a**
Total protein foods	**0·39 c**	**0·63 c**	**0·37 c**	0·11	0·11	**0·24 a**	**0·59 c**	**0·20 a**	–0·04	0·18
Seafood and plant proteins	**0·23 a**	**0·64 c**	**0·21 a**	0·01	0·10	0·10	**0·58 c**	0·09	0·09	0·11
Fatty acids‡	**0·37 c**	**0·54 c**	0·10	**0·28 b**	0·14	**0·23 a**	**0·53 c**	**0·30 b**	0·01	0·02
Refined grains	**0·55 c**	**0·49 c**	0·13	**0·68 c**	**0·25 b**	**0·54 c**	**0·55 c**	**0·24 a**	**0·70 c**	**0·37 c**
Na	**0·44 c**	**0·59 c**	**0·35 c**	**0·28 b**	–0·06	**0·27 b**	**0·41 c**	**0·42 c**	**0·33 c**	–0·09
Added sugars	**0·29 b**	**0·34 c**	**0·38 c**	0·12	**0·33 c**	**0·28 b**	**0·55 c**	**0·37 c**	–0·07	**0·27 b**
Saturated fats	**0·41 c**	**0·57 c**	**0·21 a**	0·18	**0·36 c**	**0·28 b**	**0·44 c**	0·17	**0·26 b**	**0·22 a**
NRF9.3§	**0·49 c**	**0·50 c**	**0·42 c**	**0·52 c**	**0·34 c**	**0·57 c**	**0·65 c**	**0·44 c**	**0·45 c**	**0·26 b**
Protein	–0·01	0·12	–0·08	**0·56 c**	**0·38 c**	0·17	**0·36 c**	0·18	**0·25 b**	**0·35 c**
Dietary fibre	**0·54 c**	**0·45 c**	**0·39 c**	**0·45 c**	0·11	**0·60 c**	**0·45 c**	**0·36 c**	**0·49 c**	**0·22 a**
Vitamin A	**0·47 c**	**0·32 c**	**0·33 c**	**0·35 c**	**0·36 c**	**0·45 c**	**0·52 c**	**0·31 a**	**0·35 c**	**0·19 a**
Vitamin C	**0·31 b**	**0·46 c**	**0·27 b**	**0·27 b**	0·13	**0·56 c**	**0·57 c**	**0·25 b**	**0·22 a**	**0·33 c**
Vitamin D	**0·26 b**	**0·33 c**	0·13	**0·21 a**	**0·33 c**	**0·21 a**	0·18	0·18	**0·20 a**	**0·23 a**
Ca	**0·50 c**	**0·39 c**	0·17	**0·42 c**	**0·19 a**	**0·44 c**	**0·53 c**	**0·33 c**	**0·21 a**	0·17
Fe	**0·84 c**	**0·68 c**	**0·64 c**	**0·75 c**	**0·22 a**	**0·46 c**	**0·59 c**	**0·24 b**	**0·42 c**	**0·24 a**
K	**0·52 c**	**0·33 c**	**0·33 c**	**0·42 c**	0·09	**0·56 c**	**0·53 c**	**0·29 b**	**0·50 c**	**0·27 b**
Mg	**0·41 c**	**0·34 c**	**0·36 c**	**0·48 c**	**0·27 b**	**0·50 c**	**0·49 c**	**0·36 c**	**0·46 c**	**0·42 c**
Added sugars	**0·31 b**	**0·42 c**	**0·36 c**	–0·00	**0·36 c**	**0·31 b**	**0·51 c**	**0·28 b**	0·03	**0·30 b**
Saturated fats	**0·45 c**	**0·59 c**	0·08	**0·26 b**	**0·37 c**	**0·41 c**	**0·46 b**	**0·25 b**	**0·30 b**	**0·29 b**
Na	**0·59 c**	**0·63 c**	**0·41 c**	**0·31 c**	–0·04	**0·34 c**	**0·40 c**	**0·48 c**	**0·34 c**	–0·08
Energy intake (MJ/d)	**0·38 c**	**0·58 c**	**0·31 b**	**0·31 b**	**0·44 c**	**0·39 c**	**0·65 c**	**0·45 c**	**0·44 c**	**0·46 c**
Percentage of energy intake	–	**0·59 c**	**0·27 b**	**0·40 c**	**0·44 c**	–	**0·63 c**	**0·45 c**	**0·47 c**	**0·49 c**

* Values are Spearman correlation coefficients (a: *P* < 0.05, b: *P* < 0.01, and c: *P* < 0.001; shown in bold). For the whole grains component in snacks, Pearson correlation coefficients were not available because all the participants were non-consumers in the MDHQ.

† Calculated as the sum of all components scores. A maximum score is 100. A maximum score for each component is as follows: 5 for total fruits, whole fruits, total vegetables, greens and beans, total protein foods, and seafood and plant proteins and 10 for whole grains, dairy products, fatty acids, refined grains, Na, added sugars and saturated fats. A higher score indicates a higher diet quality (i.e. a lower intake for refined grains, Na, added sugars, and saturated fats components and a higher intake for other components).

‡ Defined as the ratio of the sum of PUFA and MUFA to SFA.

§ Calculated as the sum of scores for nine nutrients to encourage (i.e. protein, dietary fibre, vitamins A, C and D, Ca, Fe, K and Mg) minus the sum of scores for three nutrients to limit (i.e. added sugars, saturated fats and Na). A maximum score is 900. For each component, a maximum score is 100, except for added sugars, saturated fats and Na components, for which a maximum score is infinite depending on the intake level. A higher score indicates a higher diet quality, except for added sugars, saturated fats and Na components, for which a higher score indicates an unfavourable dietary intake (i.e. higher intakes of added sugars, saturated fats and Na).

#### Bland–Altman plots

**Fig. 2.**
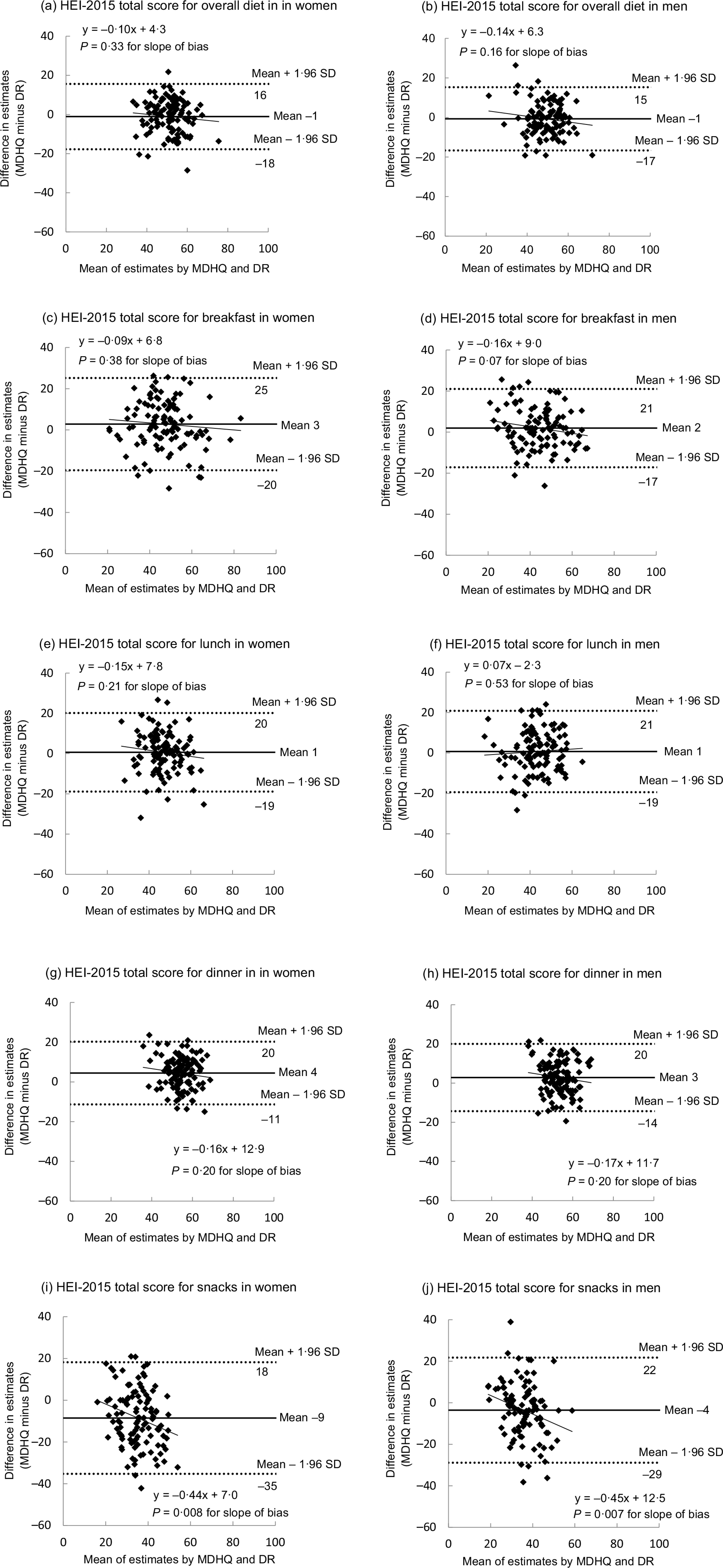
Bland–Altman plots assessing the agreement between estimates of the Healthy Eating Index-2015 (HEI-2015) total score derived from the 4-d weighed dietary record (DR) and those derived from the web version of the Meal-based Diet History Questionnaire (MDHQ) in 111 Japanese women (a: overall diet, c: breakfast, e: lunch, g: dinner and i: snacks) and 111 Japanese men (b: overall diet, d: breakfast, f: lunch, h: dinner and j: snacks), according to meal type.

Figure 2 shows Bland–Altman plots assessing the agreement between estimates of the HEI-2015 total score derived from the DR and those derived from the web MDHQ, according to the meal type. As mentioned above, the mean difference (MDHQ − DR) was relatively small in any analysis, with a range of −9 (snacks in women) to +4 (dinner in women). Regardless of sex and meal type, the limits of agreement (mean difference plus-minus 1·96 standard deviation of the difference) were generally wide, indicating poor to moderate agreement at the individual level. There was no indication of proportional bias between the web MDHQ and DR, except for snacks in both sexes, in which the HEI-2015 total score tended to be underestimated by the web MDHQ as the average score increased.

**Fig. 3.**
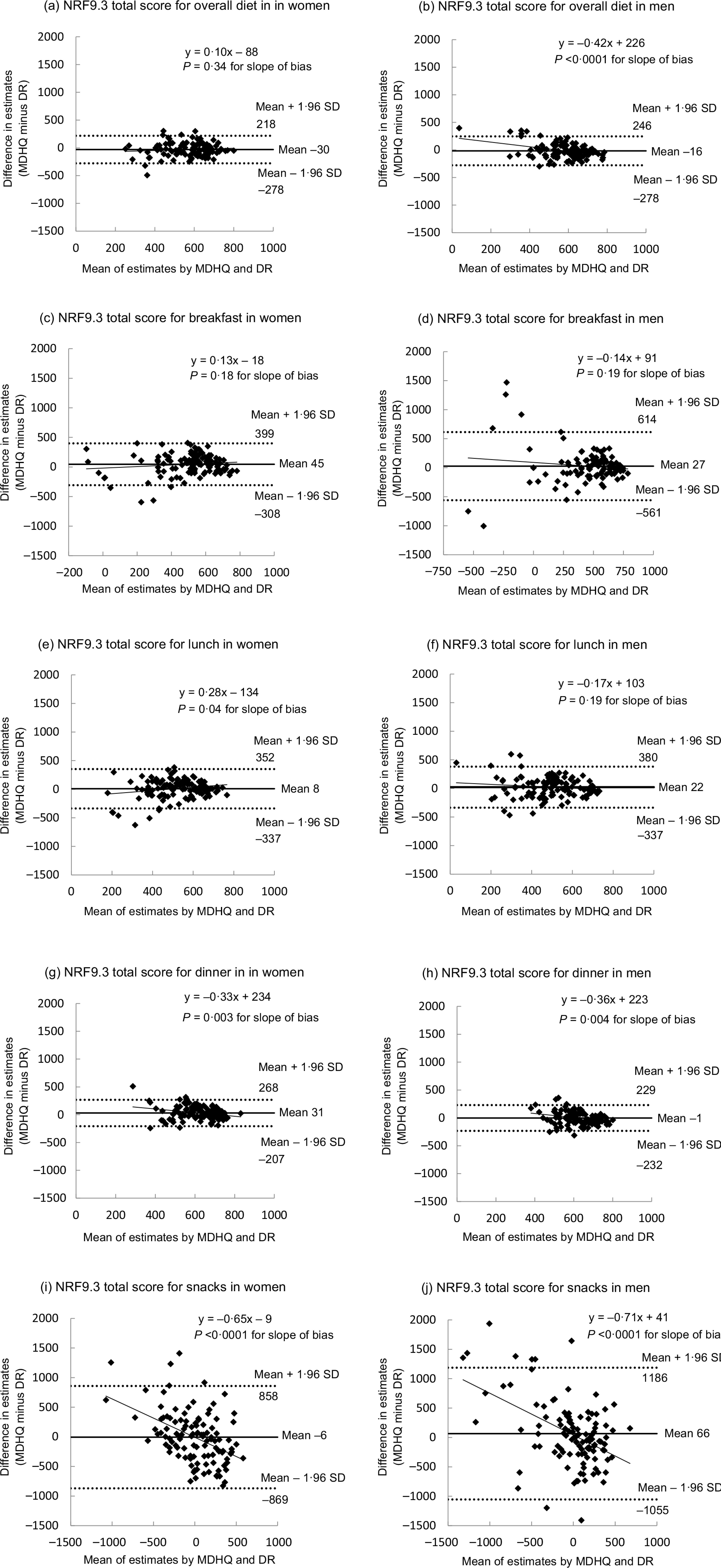
Bland–Altman plots assessing the agreement between estimates of the Nutrient-Rich Food Index 9.3 (NRF9.3) total score derived from the 4-d weighed dietary record (DR) and those derived from the web version of the Meal-based Diet History Questionnaire (MDHQ) in 111 Japanese women (a: overall diet, c: breakfast, e: lunch, g: dinner and i: snacks) and 111 Japanese men (b: overall diet, d: breakfast, f: lunch, h: dinner and j: snacks), according to meal type.

Bland–Altman plots for the NRF9.3 total score (Fig. 3) generally provided similar findings. The mean difference (MDHQ − DR) was again relatively small in any analysis, with a range of –30 (overall diet in women) to +66 (snacks in men). Regardless of sex and meal type, the limits of agreement were generally wide, indicating poor to moderate agreement at the individual level. Furthermore, with some exceptions, there was an indication of proportional bias between the web MDHQ and DR. The NRF9.3 total scores for overall diet (men only), dinner (both sexes) and snacks (both sexes) tended to be overestimated by the web MDHQ as the average score decreased, while the total score for lunch in women tended to be underestimated by the web MDHQ as the average score decreased.

### Results on the paper version of Meal-based Diet History Questionnaire

Identical analyses of the paper MDHQ were conducted (online Supplementary Table S2 for median estimations, online Supplementary Table S3 for Spearman correlations, online Supplementary Fig. S2 for Bland–Altman plots for the HEI-2015 total score and online Supplementary Fig. S3 for Bland–Altman plots for the NRF9.3 total score). The results for the paper MDHQ were generally similar to those for the web MDHQ, except for somewhat high Spearman correlation coefficients between the paper MDHQ and the DR.

## Discussion

To our knowledge, this is the first study to examine the relative validity of the MDHQ, a novel, purpose-built, dedicated dietary assessment questionnaire to collect data on dietary intake at each meal type, for assessing the overall diet quality and quality of each meal type. Using the 4-d weighed DR as a reference method, this study showed that both the web and paper versions of the MDHQ had an acceptable ability for ranking individuals according to the quality of overall diet, breakfast, lunch and dinner (but not snacks) as assessed using the HEI-2015 and NRF9.3. In contrast, the ability for estimating diet quality measures was generally limited, both at the group and at the individual levels.

For the median or mean estimation of overall diet quality, the present findings are broadly comparable with the results of previous relative validation study of the DHQ and BDHQ^(27)^. In both women and men (*n* 121 for each), the mean total scores of HEI-2015 estimated by the DHQ (57·3 and 54·8, respectively) or BDHQ (58·3 and 56·5, respectively) were slightly higher than those estimated by the 16-d DR (55·4 and 54·3, respectively)^(27)^. The mean total scores of NRF9.3 estimated by the DHQ were lower than that by the DR in both women (675 *v*. 704) and men (674 *v*. 728), while that estimated by the BDHQ (759) was higher than that by the DR in women, with no difference in men (740)^(27)^.

For the ability to rank individuals in a population, the Pearson correlation coefficient of the HEI-2015 total score estimated by the DHQ and BDHQ with that by the DR was 0·57 and 0·52 in women, respectively, and 0·51 and 0·43 in men, respectively^(27)^. The Pearson correlation coefficient with regard to NRF9.3 total score was 0·61 for both the DHQ and BDHQ in women and 0·55 for the DHQ and 0·37 for the BDHQ in men^(27)^. Only a few other studies have examined the validity (ranking ability) of dietary assessment questionnaires using other diet quality measures. For example, the Pearson correlation coefficient between the Diet Quality Index Revised estimated by an FFQ and that estimated by 2 × 7-d DR was 0·66 in 127 US men^(46)^. For a modified Mediterranean diet score and a Mediterranean-like diet score calculated from an FFQ, the Pearson correlation coefficients with those derived from 10 or more 24-h dietary recalls were 0·48 and 0·62, respectively, in 107 Spanish adults^(47)^. The Pearson correlation coefficient between the Dutch Healthy Diet Index calculated using an FFQ and that calculated using 2 × 24-h dietary recalls was 0·48 in 121 adults^(48)^. For a diet quality score assessing the compliance with the American Cancer Society dietary guidelines for cancer prevention, the Pearson correlation coefficient between an FFQ and 4 or more 24-h dietary recalls was 0·65 for 244 men and 0·54 for 433 women^(49)^. In a US study, the Spearman correlation coefficients between six diet quality scores (including the Alternate Healthy Eating Index-2010 and the Dietary Approaches to Stop Hypertension Trial score) derived from an FFQ and those derived from 2 × 7-d DR ranged from 0·43 to 0·66 in 652 men and from 0·47 to 0·67 in 742 women^(50)^. Taken together, the present study suggests that the MDHQ’s ability for ranking individuals according to a measure of overall diet quality is not inferior to that of the FFQ mentioned above, as well as the DHQ and BDHQ.

We are unaware of previous studies in which the validity of diet quality (and energy intake variables) for each meal type was assessed. Generally, we found that the level of concordance between diet quality scores derived from the MDHQ and 4-d DR was similar across all meal types based on the median intake estimation and impressions from Bland–Altman analysis, but the ability to rank individuals according to diet quality level was highest for breakfast, moderate for lunch and dinner and lowest for snacks. This finding may be due to the large between-person variability of food intake patterns at breakfast compared with that at lunch, dinner and snacks^(6,19–22)^. Alternatively, this may reflect the complex nature of lunch and dinner in terms of food consumption patterns as well as difficulty assessing snacks due to their low intake^(6,19–22)^. To partially support this finding, the median Pearson correlation coefficient between energy intake from twelve food groups estimated using an FFQ and a 7-d DR in a small study of Japanese adolescent girls (*n* 63) was higher at breakfast (0·71) than that at lunch (0·38) and dinner (0·44); this FFQ was not designed to assess the snack intake^(51)^. Similar results were also observed in a small sample of Japanese adults (twenty-nine men and sixty women)^(52)^. Nevertheless, given that the results on energy intake are also satisfactory as well as the lack of this kind of dietary assessment tool, the present findings generally support the appropriateness of the MDHQ for assessing meal-specific diet quality, in addition to overall diet quality.

Irrespective of sex, meal type and diet quality score, the Bland–Altman plots showed poor to moderate agreement between the MDHQ and DR. This is generally consistent with a previous study in Spain mentioned above^(47)^ and our previous study of the DHQ and BDHQ^(27)^. Thus, the absolute score of HEI-2015 and NRF9.3 should be interpreted with considerable caution, particularly at the individual level.

In this study, the findings for the web MDHQ were generally similar to those for the paper MDHQ, although the Spearman correlation coefficients with the DR were somewhat high for the paper MDHQ compared with that for the web MDHQ. This is not surprising given that the paper MDHQ was completed after conducting the DR, while the web MDHQ was completed before conducting the DR. While online questionnaires are preferred for administrative and cost purposes, in the real-world settings, not all study participants may be willing to complete the online questionnaires. Thus, a direct comparison between the web and paper versions of the MDHQ is warranted to assess the comparability or compatibility of these two modes but is beyond the scope of this study.

Several limitations in the present study warrant mention. First, whereas the present study was conducted in diverse regions (fourteen of forty-seven prefectures), the present population consisted of volunteers, not a nationally representative sample of the Japanese population. The participants may have been biased towards greater health consciousness, higher socio-economic status or both. For example, the education level in the present population was higher than that in a national representative sample of women (55·9 % completed junior high school or high school, 27·6 % completed college or technical school and 15·6 % completed a university degree or higher) and men (52·9, 12·9 and 33·7 %, respectively)^(53)^. However, the mean HEI-2015 for overall diet derived from the DR in the present population (51·6 for women and 49·4 for men) was somewhat lower than that in a national representative sample (52·9 for women and 51·3 for men)^(54)^, which appears to be mainly due to lower fruit intake in the present population. Meanwhile, the prevalence of current smokers and mean values of body height, body weight and BMI in the present participants^(23)^ were similar to those in a nationally representative sample (women: 7·6 %, 154·3 (sd 6·7) cm, 53·6 (sd 9·2) kg and 22·5 (sd 3·7) kg/m^2^, respectively; men: 27·1 %, 167·7 (sd 6·9) cm, 67·4 (sd 12·0) kg and 23·9 (sd 3·6) kg/m^2^, respectively)^(55)^. Ideally, further validation should be conducted using a more representative sample.

Second, the weighed DR, a reference method in this study, is susceptible to measurement errors due to the erroneous recording and potential changes in eating behaviour^(14)^. However, the weighed DR is the first method of choice for validating the dietary assessment questionnaires because the errors in weighed DR are thought to be less correlated with those in dietary assessment questionnaires compared with the errors in 24-h dietary recall or other instruments that rely on memory^(14)^. Additionally, although the dietary recording period was set to 4 d (to avoid lower participants motivation and even alteration of dietary habits potentially caused by a long-term DR^(56)^), this duration might not be sufficient for capturing estimates of habitual intake. Considering that increasing the number of recording days in the reference method improves the apparent validity of a dietary assessment questionnaire^(14,57)^, efforts to increase the duration of recording in the reference method would be important in future validation studies.

Finally, because the data collection was conducted over a narrow time frame (between August and October 2021; late summer and early autumn in Japan) as well as due to the 1-month time reference period used in the MDHQ, potential seasonal differences in dietary intake^(58–60)^ and thus in the validity of the MDHQ could not be considered in the present study. However, the results of our previous validation study of the DHQ and BDHQ suggested that a single administration of a questionnaire assessing the dietary habits during the previous month may reasonably capture the habitual dietary intake over a longer period (i.e. 1 year)^(27,34,35,61)^. There is no strong reason to consider that the MDHQ is an exception in this regard.

In conclusion, compared with the 4-d DR, both the web and paper versions of the MDHQ showed an acceptable ability for ranking individuals according to the quality of overall diet, breakfast, lunch and dinner (but not snacks) as assessed using the HEI-2015 and NRF9.3. In contrast, the ability of the MDHQ for estimating diet quality measures was generally limited, both at the group and at the individual levels. Taken together, we consider that the MDHQ, a novel, purpose-built, dedicated dietary assessment questionnaire to collect data on dietary intake at each meal type, might be useful for future nutritional epidemiological research on diet–disease relationships, not only for focusing on overall diet but also with a particular focus on meal quality, meal patterns and time of day of dietary intake, or chrono-nutrition research. Nevertheless, both the strengths and disadvantages of the MDHQ described in this study should be carefully considered in any setting.

## Supplementary material

For supplementary material accompanying this paper visit https://doi.org/10.1017/S000711452200352X. click here to view supplementary material
