# Supplementary material for: Relative validity of the online Meal-based Diet History Questionnaire for evaluating the overall diet quality and quality of each meal type in Japanese adults
Source: Br J Nutr. 2022 Nov 3;130(4):679–93. doi: 10.1017/S000711452200352X (PMC10357320; doi:10.1017/S000711452200352X)
Supplement: Supplementary file 1 [file S000711452200352Xsup001.pdf]

Supplementary Table S1. List of food groups estimated from the Meal-based Diet History Questionnaire

| Tier 1 food group | Tier 2 food group |
|---|---|
| Rice | White rice; brown rice |
| Bread | White bread; wholegrain bread |
| Noodles | Wheat noodles; Chinese noodles; instant noodles; spaghetti; buckwheat noodles |
| Miso soup | --- |
| Potatoes | --- |
| Pulses and nuts | Soy milk; tofu (i.e., soybean curd); natto (i.e., fermented soybeans); tofu products; peanuts and nuts; all other pulses and nuts |
| Vegetables | Edamame (i.e., immature soybeans) and peas; seaweeds; pumpkins; mushrooms; cabbage; cucumbers; bitter melon; burdock; radishes; onions; Chinese cabbage; tomatoes; eggplants; carrots; green peppers; broccoli; green leafy vegetables; bean sprouts; lettuce; all other vegetables |
| Pickled vegetables | --- |
| Fruits | Strawberries; persimmons; citrus; kiwi fruit; watermelon; pears; bananas; grapes; melon; peaches; apples; all other fruit |
| Fish and shellfish | Oily fish; red meat fish; squid and octopus; eel; shrimp and crab; shellfish; small fish with bones; fish eggs; dried fish; salmon; white meat fish; ground fish meat products; canned tuna; all other fish and shellfish |
| Meat | Liver; processed meat; beef; chicken; pork; all other meat |
| Eggs | --- |
| Dairy products | Ice cream; cheese; low-fat milk; yogurt; full-fat milk; all other dairy products |
| Confectioneries | Candies, caramels, and chewing gum; Japanese bread with a sweet filling; snacks made from wheat flour; jellies; rice crackers; chocolates; biscuits and cookies; cakes; Japanese sweets |
| Fruit and vegetable juice | --- |
| Alcoholic beverages | Beer; sake; shochu (i.e., Japanese distilled beverages); wine; whiskey and other spirits |
| Soft drinks | --- |
| Green tea | --- |
| Barley tea | --- |
| Oolong tea | --- |
| Black tea | --- |
| Coffee | --- |
| Water | --- |
| Breakfast cereals | --- |
| Seasonings | Sugar added to coffee and tea; salt for cooking; vegetable oils; sugar for cooking; salt for soup; soy sauce; jam for bread; fat spread for bread; mayonnaise and dressing |

Supplementary Table S2. Median estimates of the total and component scores of Healthy Eating Index-2015 (HEI-2015) and Nutrient-Rich Food Index 9.3 (NRF9.3), energy intake, and percentage of energy intake derived from the paper version of the Meal-based Diet History Questionnaire (MDHQ) in 111 Japanese women and 111 Japanese men, according to meal type†

| | Women | | | | | | | | | | Men | | | | | | | | | |
|---|---|---|---|---|---|---|---|---|---|---|---|---|---|---|---|---|---|---|---|---|
| | Overall diet | | Breakfast | | Lunch | | Dinner | | Snacks | | Overall diet | | Breakfast | | Lunch | | Dinner | | Snacks | |
| | Median | P25, P75 | Median | P25, P75 | Median | P25, P75 | Median | P25, P75 | Median | P25, P75 | Median | P25, P75 | Median | P25, P75 | Median | P25, P75 | Median | P25, P75 | Median | P25, P75 |
| HEI-2015‡ | 51.4 | 45.2, 56.7 | **50.4 c** | 42.4, 56.2 | 47.5 | 43.3, 52.3 | **57.4 c** | 51.5, 61.2 | **34.1 c** | 26.7, 41.3 | 50.3 | 44.9, 55.8 | **47.8 a** | 39, 52.8 | **46.2 a** | 40.1, 51.6 | **55.6 c** | 51.3, 59.2 | 36.9 | 30.2, 43.9 |
| Total fruits | **2.0 c** | 0.8, 3.6 | 1.9 | 0.3, 4.1 | 0.7 | 0, 2.2 | **0.7 c** | 0, 2.4 | **3.4 c** | 0.2, 5.0 | **1.2 c** | 0.3, 2.9 | **1.0 a** | 0, 2.8 | **0.1 b** | 0, 1.4 | **0.4 c** | 0, 1.4 | **2.6 c** | 0.1, 5.0 |
| Whole fruits | **3.2 b** | 1.4, 5.0 | **2.6 a** | 0.4, 5.0 | 1.2 | 0, 3.5.0 | **1.2 c** | 0, 4.7 | **4.2 c** | 0.2, 5.0 | **1.5 c** | 0.5, 4.3 | **1.3 a** | 0, 4.4 | **0.1 a** | 0, 1.6 | **0.8 c** | 0, 2.6 | **2.1 c** | 0, 5.0 |
| Total vegetables | 5.0 | 4.3, 5.0 | **3.6 c** | 1, 5.0 | 4.8 | 2.1, 5.0 | 5.0 | 5.0, 5.0 | **0.5 a** | 0.2, 1.2 | **4.9 a** | 3.4, 5.0 | **2.9 a** | 0.1, 5.0 | 3.7 | 1.8, 5.0 | **5.0 a** | 4.9, 5.0 | **0.5 c** | 0.1, 2.1 |
| Greens and beans | **2.5 b** | 1.2, 4.0 | **1.6 c** | 0.2, 3.1 | 1.5 | 0.6, 2.8 | **4.2 c** | 2.0, 5.0 | **0.3 c** | 0.1, 0.5 | **1.8 a** | 1.1, 2.9 | **0.9 b** | 0, 2.1 | 1.1 | 0.3, 2.1 | **2.7 c** | 1.5, 5.0 | **0.1 c** | 0, 0.3 |
| Whole grains | **0.7 c** | 0.1, 1.5 | **1.6 c** | 0, 3.9 | **0.5 c** | 0, 1.6 | **0 c** | 0, 0.5 | 0 | 0, 0 | **0.2 c** | 0, 1.3 | **0.2 c** | 0, 3.2 | **0 b** | 0, 1.5 | **0 c** | 0, 0.3 | 0 | 0, 0 |
| Dairy | 2.6 | 1.6, 4.4 | **5.1 c** | 2.0, 10 | 0.9 | 0.2, 2.2 | **0.5 a** | 0, 1.8 | 2.3 | 0.8, 5.3 | 1.1 | 0.4, 2.6 | 2.3 | 0.1, 6.7 | 0.1 | 0, 1.0 | 0.3 | 0, 0.8 | 0.8 | 0.3, 2.4 |
| Total protein foods | **4.8 c** | 4.1, 5.0 | 4.4 | 2.5, 5.0 | 4.6 | 3.1, 5.0 | 5.0 | 5.0, 5.0 | **0.3 b** | 0.1, 0.6 | **4.7 c** | 4, 5.0 | 4.3 | 2.2, 5.0 | 4.6 | 3.2, 5.0 | 5.0 | 5.0, 5.0 | **0.1 a** | 0, 0.4 |
| Seafood and plant proteins | 5.0 | 5.0, 5.0 | **5.0 a** | 0.3, 5.0 | 4.6 | 2.3, 5.0 | **5.0 a** | 5.0, 5.0 | 0.4 | 0.2, 0.8 | 5.0 | 4.7, 5.0 | 4.3 | 0, 5.0 | 5.0 | 1.8, 5.0 | 5.0 | 5.0, 5.0 | 0.2 | 0, 0.5 |
| Fatty acids§ | 5.6 | 3.5, 8.2 | 4.1 | 1.0, 7.9 | 7.4 | 5.1, 10 | **10 c** | 10, 10 | **0 c** | 0, 0 | 6.4 | 4.6, 8.8 | 4.2 | 2.2, 8.5 | 7.8 | 4.6, 10 | **10 c** | 9.6, 10 | **0 a** | 0, 0 |
| Refined grains | 1.0 | 0, 3.3 | 0 | 0, 2.3 | **0 b** | 0, 0 | **2.7 b** | 0.5, 8.5 | **5.1 c** | 1.7, 9.0 | 0.2 | 0, 2.8 | 0 | 0, 0.9 | 0 | 0, 0 | **2.4 a** | 0, 8.1 | **9.2 b** | 4.6, 10 |
| Sodium | **0 c** | 0, 0.7 | **1.8 c** | 0, 6.5 | 0 | 0, 2.5 | **0 c** | 0, 0 | 10 | 10, 10 | **0 c** | 0, 2.7 | **0.5 c** | 0, 6.3 | 0 | 0, 4.1 | **0 a** | 0, 2.9 | **10 b** | 10, 10 |
| Added sugars | **9.5 b** | 7.9, 10 | 9.9 | 8.0, 10 | 10 | 9.8, 10 | 10 | 10, 10 | **0 c** | 0, 2.6 | 10 | 8.7, 10 | 10 | 8.0, 10 | 10 | 10, 10 | 10 | 10, 10 | **1.0 a** | 0, 6.2 |
| Saturated fats | 9.1 | 7.2, 10 | 7.9 | 4.7, 10 | **10 a** | 8.6, 10 | **10 c** | 9.3, 10 | 3.9 | 0, 9.3 | **10 c** | 9.5, 10 | **8.7 a** | 6.7, 10 | **10 c** | 10, 10 | **10 c** | 10, 10 | 9.3 | 3.6, 10 |
| NRF9.3‖ | **581 b** | 472, 656 | **596 c** | 451, 668 | 558 | 427, 629 | **660 c** | 584, 721 | 26 | −124, 189 | 614 | 525, 668 | **562 a** | 429, 646 | **544 b** | 447, 626 | 645 | 575, 701 | **136 a** | −88, 311 |
| Protein | 100 | 100, 100 | **100 a** | 100, 100 | **100 a** | 100, 100 | 100 | 100, 100 | 72 | 62, 89 | 100 | 100, 100 | 100 | 100, 100 | 100 | 100, 100 | **100 b** | 100, 100 | 68 | 51, 79 |
| Dietary fiber | 77 | 65, 87 | 81 | 63, 97 | 74 | 60, 87 | **93 a** | 71, 100 | **50 b** | 40, 61 | 73 | 57, 82 | 82 | 63, 97 | 69 | 57, 83 | 80 | 55, 99 | **48 c** | 26, 67 |
| Vitamin A | **69 c** | 54, 87 | **67 c** | 52, 87 | 60 | 29, 82 | **87 c** | 53, 100 | 43 | 30, 59 | **55 a** | 39, 72 | **55 b** | 33, 78 | 47 | 26, 68 | **65 b** | 40, 92 | **34 c** | 14, 54 |
| Vitamin C | 88 | 64, 100 | **81 c** | 38, 100 | 74 | 44, 95 | 100 | 88, 100 | **63 b** | 18, 100 | 100 | 74, 100 | **84 a** | 33, 100 | 77 | 41, 100 | 100 | 80, 100 | **100 c** | 29, 100 |
| Vitamin D | 64 | 49, 88 | **60 c** | 40, 90 | **49 c** | 34, 72 | **82 b** | 65, 100 | **19 a** | 12, 25 | 84 | 62, 100 | **76 c** | 46, 100 | **60 c** | 36, 93 | **100 b** | 74, 100 | 15 | 5, 25 |
| Calcium | **89 c** | 75, 100 | **100 c** | 86, 100 | 63 | 47, 84 | **70 b** | 56, 89 | 98 | 73, 100 | 74 | 59, 86 | **100 a** | 65, 100 | 54 | 44, 67 | 64 | 49, 77 | 98 | 66, 100 |
| Iron | 89 | 65, 100 | **86 b** | 59, 100 | **76 b** | 55, 100 | 100 | 76, 100 | 70 | 51, 97 | **100 a** | 97, 100 | 100 | 92, 100 | 100 | 85, 100 | **100 a** | 93, 100 | **86 c** | 60, 100 |
| Potassium | **100 a** | 90, 100 | **100 c** | 93, 100 | 82 | 61, 96 | 100 | 89, 100 | 100 | 85, 100 | 99 | 85, 100 | **100 a** | 87, 100 | 78 | 65, 92 | **100 a** | 81, 100 | **100 a** | 91, 100 |
| Magnesium | **100 b** | 87, 100 | **100 c** | 96, 100 | **84 a** | 69, 97 | **100 b** | 94, 100 | **94 a** | 74, 100 | **92 b** | 79, 100 | **100 b** | 82, 100 | **77 c** | 65, 89 | 97 | 80, 100 | **100 c** | 79, 100 |
| Added sugars | **52 b** | 15, 115 | 35 | 0, 110 | 0 | 0, 40 | 0 | 0, 0 | 445 | 329, 593 | 17 | 0, 83 | 2 | 0, 113 | 0 | 0, 2 | 0 | 0, 0 | 396 | 188, 612 |
| Saturated fats | 27 | 13, 50 | 39 | 17, 79 | **5 a** | 0, 32 | **10 c** | 0, 26 | 88 | 21, 136 | **6 c** | 0, 23 | **31 a** | 0, 55 | **0 c** | 0, 12 | **0 c** | 0, 10 | 25 | 0, 98 |
| Sodium | **70 c** | 53, 100 | **47 c** | 9, 113 | 77 | 42, 119 | **112 c** | 74, 141 | 0 | 0, 0 | **81 c** | 58, 110 | **70 c** | 26, 128 | 88 | 47, 131 | **93 b** | 53, 142 | **0 b** | 0, 0 |
| Energy intake (MJ/day) | **6.4 c** | 5.4, 7.2 | 1.6 | 1.2, 1.8 | **1.7 c** | 1.3, 2.0 | **2.1 c** | 1.7, 2.3 | **0.9 c** | 0.5, 1.4 | **7.9 c** | 6.7, 8.9 | **1.7 a** | 1.1, 2.1 | **2.1 c** | 1.8, 2.6 | **2.9 c** | 2.5, 3.4 | **1.1 c** | 0.4, 1.6 |
| Percentage of energy intake | --- | --- | **24.8 b** | 19.7, 29.6 | **26.9 c** | 22.4, 30.9 | **33.1 c** | 27.9, 37.2 | **14.7 c** | 9.5, 21.9 | --- | --- | **21.8 b** | 14.4, 25.4 | **27.6 c** | 23.3, 32 | **36 c** | 32.1, 43.3 | **13.6 c** | 5.6, 18.2 |

P25, 25th percentile; P75, 75th percentile.

† The values derived from the MDHQ were compared with those derived from the 4-day weighed dietary record (shown in Table 2 for women and Table 3 for men) using the

Wilcoxon signed-rank test (a: $P<0.05$, b: $P<0.01$, and c: $P<0.001$; shown in bold).
‡ Calculated as the sum of all components scores. A maximum score is 100. A maximum score for each component is as follows: 5 for total fruits, whole fruits, total vegetables, greens and beans, total protein foods, and seafood and plant proteins and 10 for whole grains, dairy products, fatty acids, refined grains, sodium, added sugars, and saturated fats. A higher score indicates a higher diet quality (i.e., a lower intake for refined grains, sodium, added sugars, and saturated fats components and a higher intake for other components).
§ Defined as the ratio of the sum of PUFA and MUFA to SFA.
|| Calculated as the sum of scores for nine nutrients to encourage (i.e., protein, dietary fiber, vitamins A, C and D, calcium, iron, potassium, and magnesium) minus the sum of scores for three nutrients to limit (i.e., added sugars, saturated fats, and sodium). A maximum score is 900. For each component, a maximum score is 100, except for added sugars, saturated fats, and sodium components, for which a maximum score is infinite depending on the intake level. A higher score indicates a higher diet quality, except for added sugars, saturated fats, and sodium components, for which a higher score indicates an unfavorable dietary intake (i.e., higher intakes of added sugars, saturated fats, and sodium).

Supplementary Table S3. Spearman correlation coefficients between estimates of the total and component scores of Healthy Eating Index-2015 (HEI-2015) and Nutrient-Rich Food Index 9.3 (NRF9.3), energy intake, and percentage of energy intake derived from the 4-day weighed dietary record and those derived from the paper version of the Meal-based Diet History Questionnaire (MDHQ) in 111 Japanese women and 111 Japanese men, according to meal type†

| | Women | | | | | Men | | | | |
|---|---|---|---|---|---|---|---|---|---|---|
| | Overall diet | Breakfast | Lunch | Dinner | Snacks | Overall diet | Breakfast | Lunch | Dinner | Snacks |
| HEI-2015‡ | **0.64 c** | **0.58 c** | **0.40 c** | **0.48 c** | 0.16 | **0.55 c** | **0.71 c** | **0.46 c** | **0.46 c** | **0.22 a** |
| Total fruits | **0.69 c** | **0.62 c** | **0.51 c** | **0.50 c** | **0.31 c** | **0.57 c** | **0.55 c** | **0.37 c** | **0.24 a** | **0.32 c** |
| Whole fruits | **0.74 c** | **0.59 c** | **0.52 c** | **0.52 c** | **0.33 c** | **0.61 c** | **0.57 c** | **0.40 c** | **0.24 a** | **0.30 c** |
| Total vegetables | **0.41 c** | **0.69 c** | **0.39 c** | **0.39 c** | **0.20 a** | **0.52 c** | **0.58 c** | **0.42 c** | **0.43 c** | 0.17 |
| Greens and beans | **0.33 c** | **0.31 b** | **0.21 a** | **0.33 c** | 0.17 | **0.29 b** | **0.36 c** | 0.12 | **0.20 a** | 0.10 |
| Whole grains | **0.44 c** | **0.43 c** | 0.12 | **0.25 b** | Not available | **0.45 c** | **0.32 c** | **0.34 c** | **0.47 c** | Not available |
| Dairy | **0.62 c** | **0.74 c** | 0.17 | **0.32 c** | **0.48 c** | **0.66 c** | **0.65 c** | **0.33 c** | **0.33 c** | **0.31 c** |
| Total protein foods | **0.27 b** | **0.49 c** | **0.35 c** | −0.05 | **0.28 b** | **0.23 a** | **0.60 c** | **0.25 b** | −0.04 | **0.27 b** |
| Seafood and plant proteins | **0.21 a** | **0.52 c** | 0.18 | −0.01 | **0.24 b** | 0.16 | **0.50 c** | **0.27 b** | 0.02 | 0.16 |
| Fatty acids§ | **0.37 c** | **0.49 c** | 0.07 | 0.10 | 0.10 | **0.25 b** | **0.56 c** | **0.26 b** | 0.10 | 0.00 |
| Refined grains | **0.56 c** | **0.39 c** | **0.37 c** | **0.66 c** | **0.34 c** | **0.64 c** | **0.54 c** | **0.36 c** | **0.74 c** | **0.39 c** |
| Sodium | **0.46 c** | **0.65 c** | **0.35 c** | **0.37 c** | −0.05 | **0.37 c** | **0.46 c** | **0.43 c** | **0.35 c** | −0.05 |
| Added sugars | **0.39 c** | **0.34 b** | **0.39 c** | 0.10 | **0.34 c** | **0.39 c** | **0.56 c** | 0.18 | −0.05 | **0.33 c** |
| Saturated fats | **0.38 c** | **0.60 c** | **0.19 a** | **0.20 a** | **0.29 b** | **0.34 c** | **0.50 c** | 0.03 | **0.26 b** | **0.53 c** |
| NRF9.3‖ | **0.65 c** | **0.55 c** | **0.49 c** | **0.62 c** | **0.34 c** | **0.56 c** | **0.63 c** | **0.54 c** | **0.44 c** | **0.41 c** |
| Protein | −0.02 | 0.12 | −0.06 | **0.56 c** | **0.48 c** | **0.25 b** | **0.40 c** | −0.07 | **0.30 b** | **0.42 c** |
| Dietary fiber | **0.62 c** | **0.50 c** | **0.51 c** | **0.47 c** | **0.25 b** | **0.66 c** | **0.37 c** | **0.45 c** | **0.56 c** | **0.34 c** |
| Vitamin A | **0.59 c** | **0.32 c** | **0.40 c** | **0.52 c** | **0.24 b** | **0.52 c** | **0.55 c** | **0.39 c** | **0.40 c** | **0.33 c** |
| Vitamin C | **0.43 c** | **0.58 c** | **0.36 c** | **0.35 c** | **0.33 c** | **0.46 c** | **0.56 c** | **0.35 c** | **0.46 c** | **0.33 c** |
| Vitamin D | **0.33 c** | **0.23 a** | **0.32 b** | **0.38 b** | **0.32 c** | **0.21 a** | **0.28 b** | **0.25 b** | 0.18 | **0.35 c** |
| Calcium | **0.60 c** | **0.46 c** | 0.19 | **0.54 c** | **0.40 c** | **0.46 c** | **0.47 c** | 0.17 | **0.33 c** | **0.40 c** |
| Iron | **0.83 c** | **0.70 c** | **0.65 c** | **0.78 c** | **0.41 c** | **0.57 c** | **0.56 c** | **0.34 c** | **0.53 c** | **0.45 c** |
| Potassium | **0.57 c** | **0.45 c** | **0.39 c** | **0.58 c** | **0.31 c** | **0.59 c** | **0.45 c** | **0.36 c** | **0.61 c** | **0.27 b** |
| Magnesium | **0.48 c** | **0.48 c** | **0.40 c** | **0.53 c** | **0.45 c** | **0.61 c** | **0.45 c** | **0.40 c** | **0.50 c** | **0.38 c** |
| Added sugars | **0.41 c** | **0.39 c** | **0.35 c** | 0.11 | **0.33 c** | **0.41 c** | **0.51 c** | 0.10 | 0.16 | **0.41 c** |
| Saturated fats | **0.44 c** | **0.61 c** | **0.20 a** | **0.23 b** | **0.29 b** | **0.39 c** | **0.53 c** | 0.03 | **0.33 c** | **0.51 c** |
| Sodium | **0.57 c** | **0.69 c** | **0.40 c** | **0.45 c** | −0.02 | **0.43 c** | **0.45 c** | **0.50 c** | **0.41 c** | −0.05 |
| Energy intake (MJ/day) | **0.34 c** | **0.49 c** | **0.29 b** | **0.32 c** | **0.54 c** | **0.46 c** | **0.73 c** | **0.47 c** | **0.47 c** | **0.60 c** |
| Percentage of energy intake | --- | **0.49 c** | **0.29 b** | **0.52 c** | **0.56 c** | --- | **0.69 c** | **0.54 c** | **0.56 c** | **0.57 c** |

† Values are expressed as Spearman correlation coefficients (a: $P$<0.05, b: $P$<0.01, and c: $P$<0.001; shown in bold). For the whole grains component, Pearson correlation coefficients were not available because all the participants were non-consumers in MDHQ.

‡ Calculated as the sum of all components scores. A maximum score is 100. A maximum score for each component is as follows: 5 for total fruits, whole fruits, total

vegetables, greens and beans, total protein foods, and seafood and plant proteins and 10 for whole grains, dairy products, fatty acids, refined grains, sodium, added sugars, and saturated fats. A higher score indicates a higher diet quality (i.e., a lower intake for refined grains, sodium, added sugars, and saturated fats components and a higher intake for other components).

§ Defined as the ratio of the sum of PUFA and MUFA to SFA.

|| Calculated as the sum of scores for nine nutrients to encourage (i.e., protein, dietary fiber, vitamins A, C and D, calcium, iron, potassium, and magnesium) minus the sum of scores for three nutrients to limit (i.e., added sugars, saturated fats, and sodium). A maximum score is 900. For each component, a maximum score is 100, except for added sugars, saturated fats, and sodium components, for which a maximum score is infinite depending on the intake level. A higher score indicates a higher diet quality, except for added sugars, saturated fats, and sodium components, for which a higher score indicates an unfavorable dietary intake (i.e., higher intakes of added sugars, saturated fats, and sodium).

(Supplementary Figure S1)

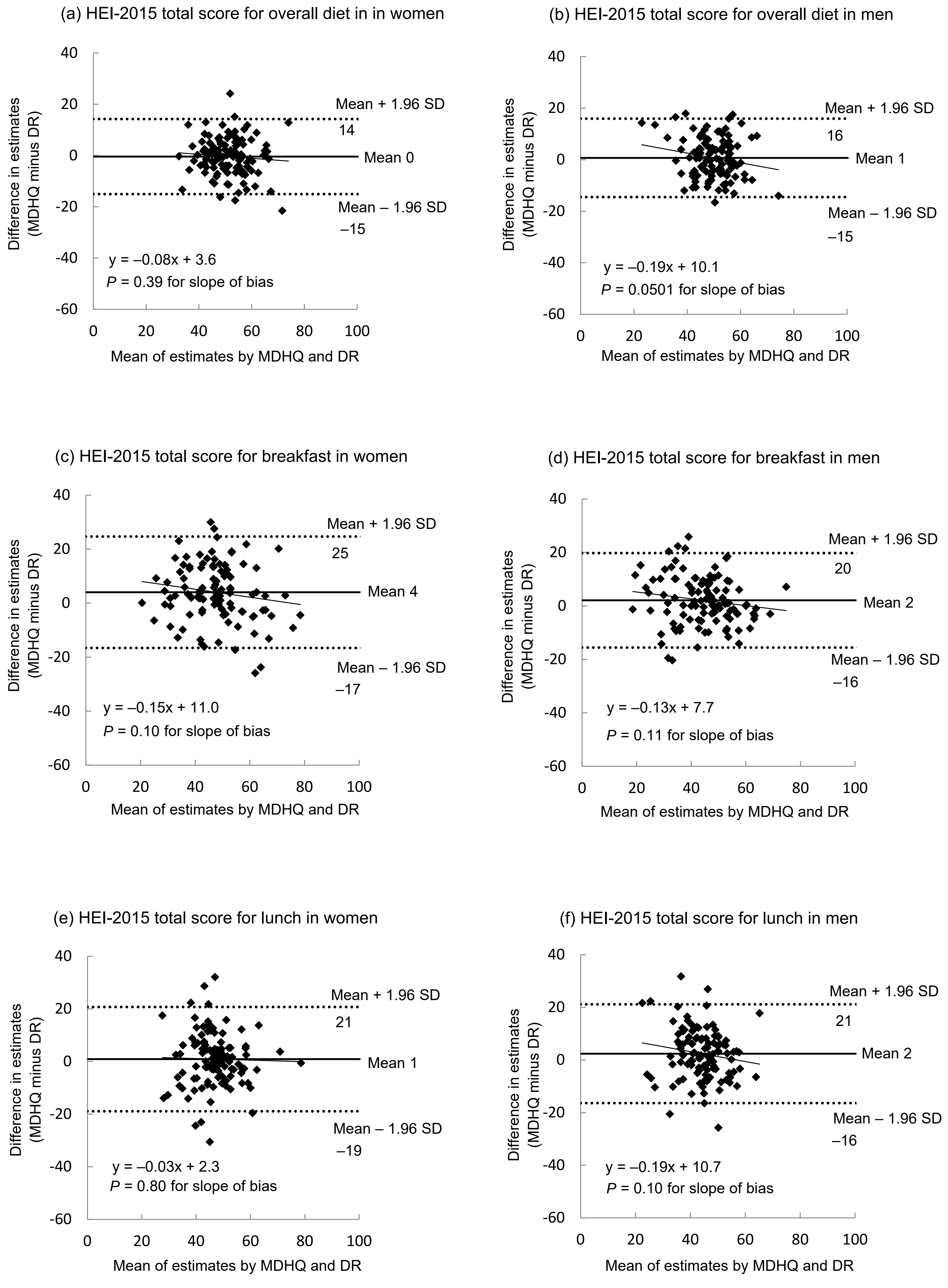

(Supplementary Figure S1)

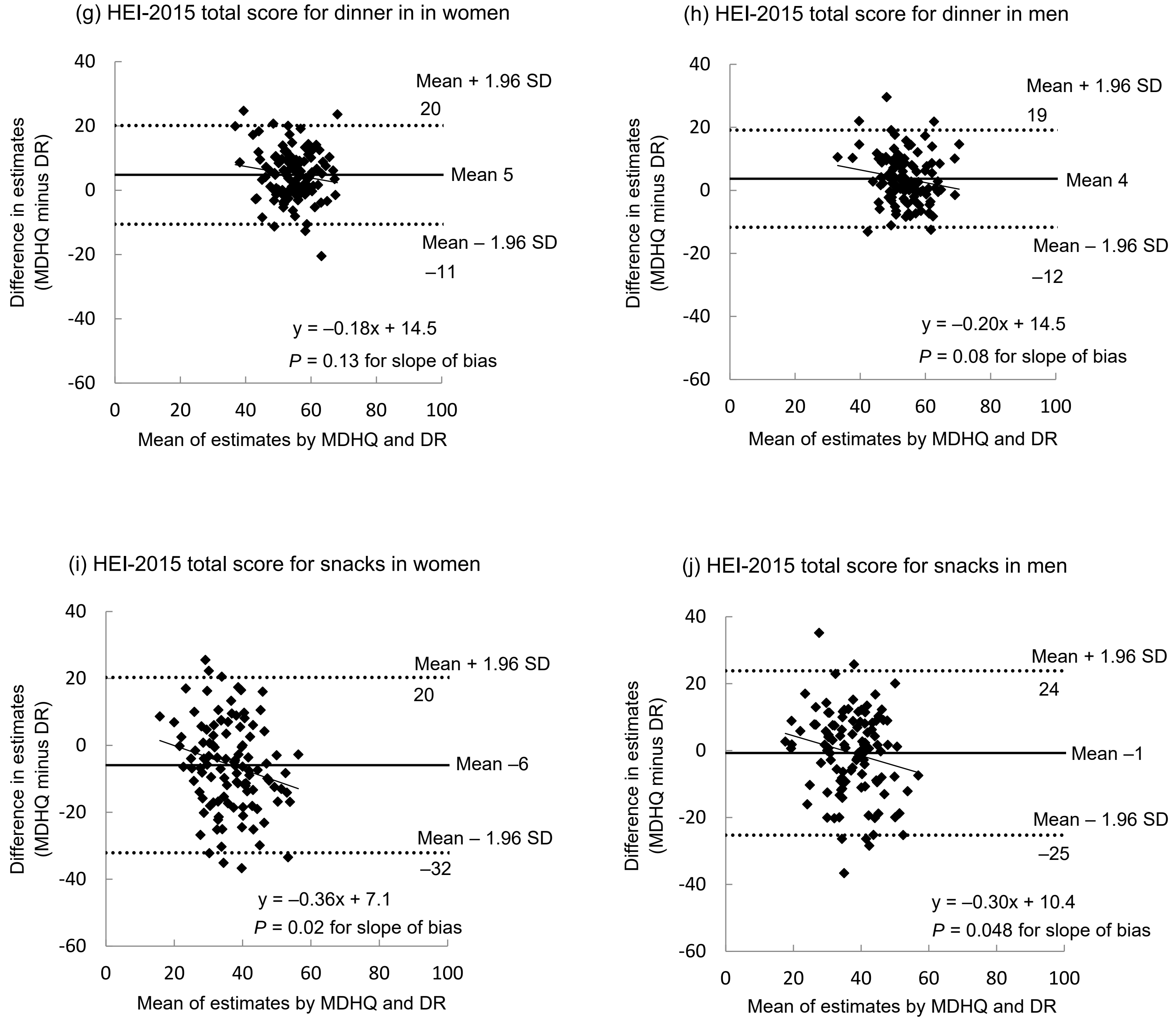


Supplementary Figure S1. Bland–Altman plots assessing the agreement between estimates of the Healthy Eating Index-2015 (HEI-2015) total score derived from the 4-day weighed dietary record (DR) and those derived from the paper version of the Meal-based Diet History Questionnaire (MDHQ) in 111 Japanese women (a: overall diet, c: breakfast, e: lunch, g: dinner, and i: snacks) and 111 Japanese men (b: overall diet, d: breakfast, f: lunch, h: dinner, and j: snacks), according to meal type

(Supplementary Figure S2)

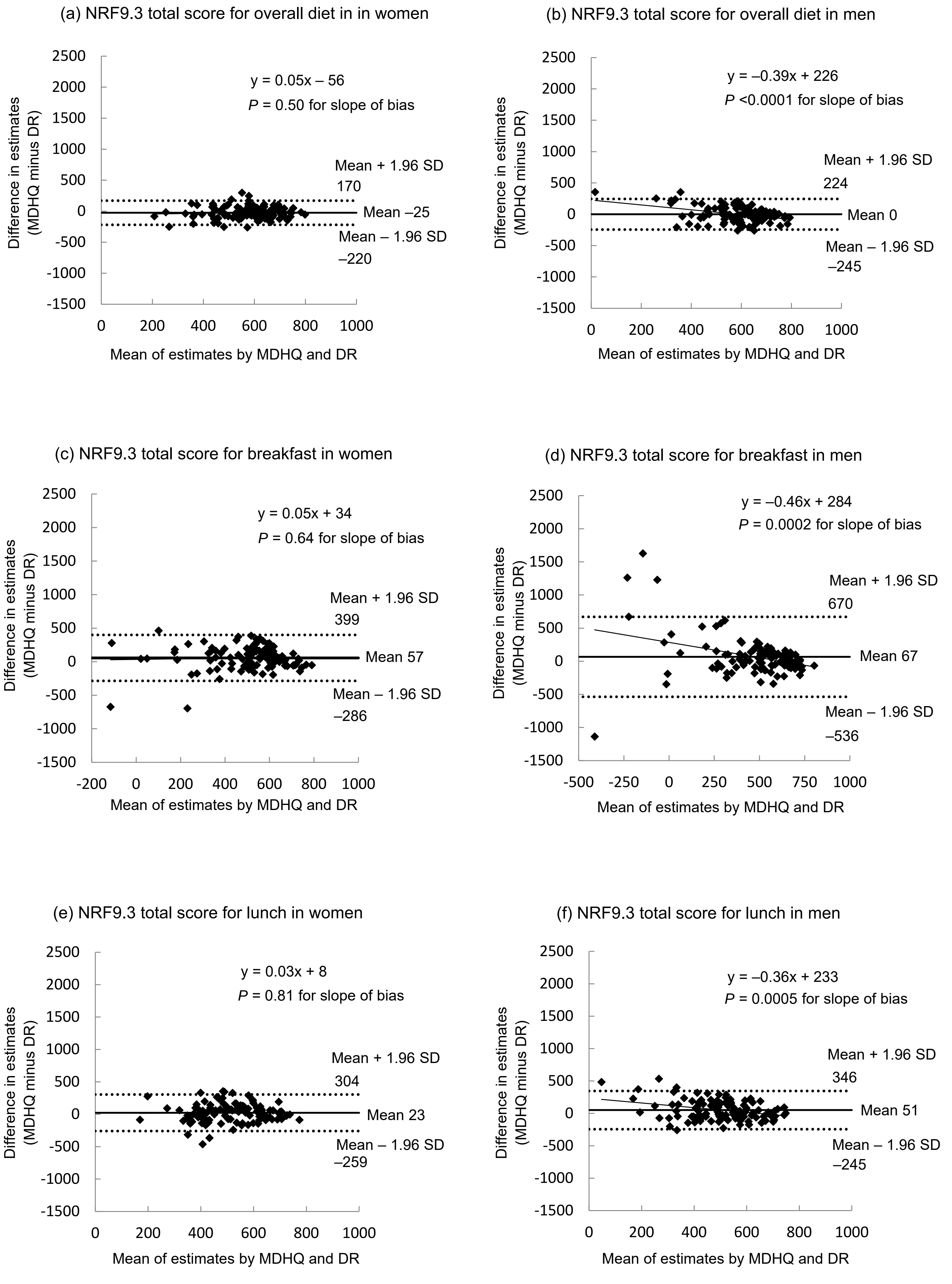

(Supplementary Figure S2)

(g) NRF9.3 total score for dinner in in women

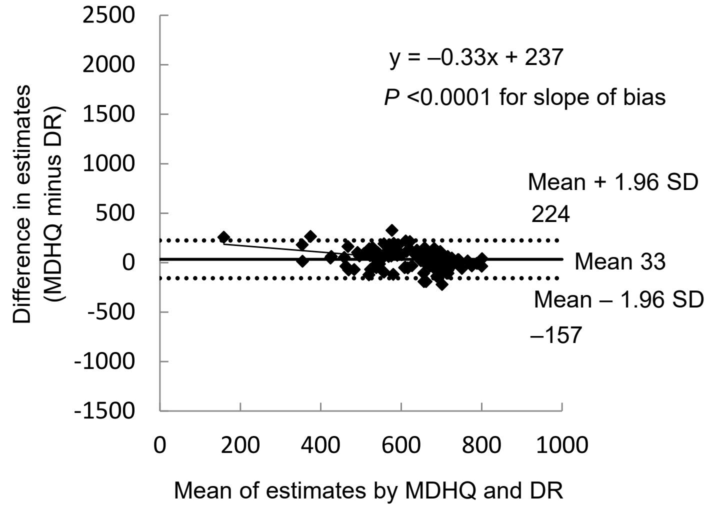


(h) NRF9.3 total score for dinner in men

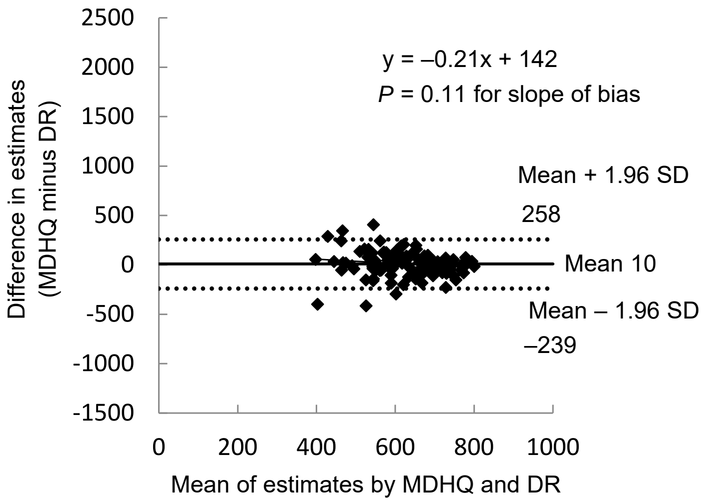


(i) NRF9.3 total score for snacks in women

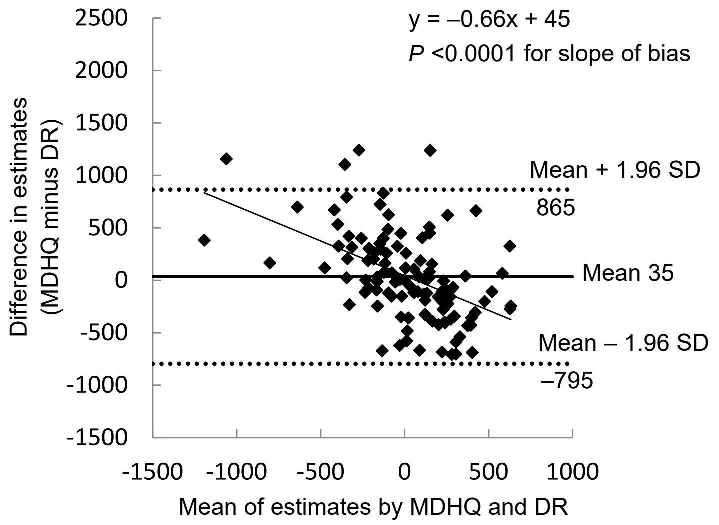


(j) NRF9.3 total score for snacks in men

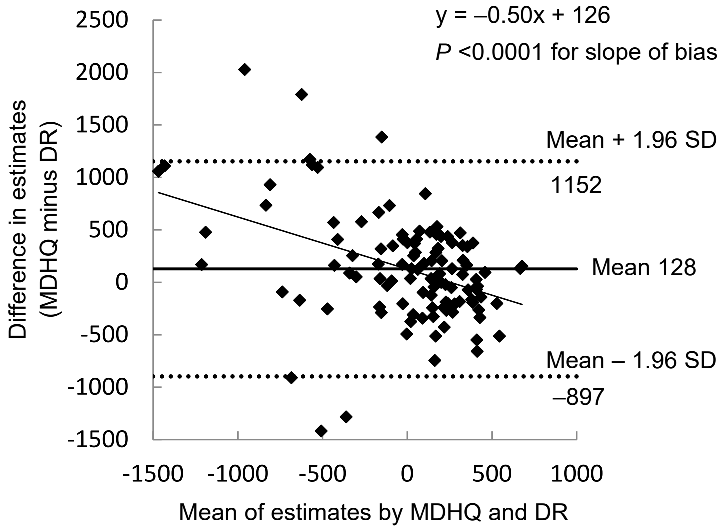


Supplementary Figure S2. Bland–Altman plots assessing the agreement between estimates of the Nutrient-Rich Food Index 9.3 (NRF9.3) total score derived from the 4-day weighed dietary record (DR) and those derived from the paper version of the Meal-based Diet History Questionnaire (MDHQ) in 111 Japanese women (a: overall diet, c: breakfast, e: lunch, g: dinner, and i: snacks) and 111 Japanese men (b: overall diet, d: breakfast, f: lunch, h: dinner, and j: snacks), according to meal type
